# Myeloid-derived suppressor cells prevent disruption of the gut barrier, preserve microbiota composition, and potentiate immunoregulatory pathways in a rat model of experimental autoimmune encephalomyelitis

**DOI:** 10.1080/19490976.2022.2127455

**Published:** 2022-10-02

**Authors:** Dušan Radojević, Marina Bekić, Alisa Gruden-Movsesijan, Nataša Ilić, Miroslav Dinić, Aleksandar Bisenić, Nataša Golić, Dragana Vučević, Jelena Đokić, Sergej Tomić

**Affiliations:** a Group for Probiotics and Microbiota-Host Interaction, Laboratory for Molecular Microbiology, Institute of Molecular Genetics and Genetic Engineering, University of Belgrade, Belgrade, Serbia; b Department for Immunology and Immunoparasitology, Institute for the Application of Nuclear Energy, University of Belgrade, Belgrade, Serbia; c Medical Faculty of the Military Medical Academy, University of Defense in Belgrade, Belgrade, Serbia

**Keywords:** Multiple sclerosis, myeloid-derived suppressor cells, microbial metabolites, gut microbiota, immunoregulatory mechanisms

## Abstract

Over-activated myeloid cells and disturbance in gut microbiota composition are critical factors contributing to the pathogenesis of Multiple Sclerosis (MS). Myeloid-derived suppressor cells (MDSCs) emerged as promising regulators of chronic inflammatory diseases, including autoimmune diseases. However, it remained unclear whether MDSCs display any therapeutic potential in MS, and how this therapy modulates gut microbiota composition. Here, we assessed the potential of *in vitro* generated bone marrow-derived MDSCs to ameliorate experimental autoimmune encephalomyelitis (EAE) in Dark Agouti rats and investigated how their application associates with the changes in gut microbiota composition. MDSCs differentiated with prostaglandin (PG)E2 (MDSC-PGE2) and control MDSCs (differentiated without PGE2) displayed strong immunosuppressive properties *in vitro*, but only MDSC-PGE2 significantly ameliorated EAE symptoms. This effect correlated with a reduced infiltration of Th17 and IFN-γ-producing NK cells, and an increased proportion of regulatory T cells in the CNS and spleen. Importantly, both MDSCs and MDSC-PGE2 prevented EAE-induced reduction of gut microbiota diversity, but only MDSC-PGE2 prevented the extensive alterations in gut microbiota composition following their early migration into Payer’s patches and mesenteric lymph nodes. This phenomenon was related to the significant enrichment of gut microbial taxa with potential immunoregulatory properties, as well as higher levels of butyrate, propionate, and putrescine in feces. This study provides new insights into the host–microbiota interactions in EAE, suggesting that activated MDSCs could be potentially used as an efficient therapy for acute phases of MS. Considering a significant association between the efficacy of MDSC-PGE2 and gut microbiota composition, our findings also provide a rationale for further exploring the specific microbial metabolites in MS therapy.

## 1. Introduction

Autoimmune diseases (AD) are steadily rising worldwide affecting 5–-10% of the global population, with Multiple Sclerosis (MS) affecting 2.8 million people worldwide, especially young individuals (20-–50 years) and women (75%).^1^ MS is a chronic inflammatory disease of the central nervous system (CNS), most commonly (85%) taking a relapse-remitting course (RRMS), resulting in progressive damage of myelin sheaths surrounding the axons, which leads to physical, sensory, and neurological dysfunction.^2^ Mechanisms of the disease involve the migration of T helper (Th)1 and Th17 cells across the blood–brain barrier (BBB) and production of proinflammatory cytokines in the CNS.^3^ Activated myeloid cells are considered key initiators of MS causing a brake of tolerogenic mechanisms.^4^ Although the underlying cause of MS remains elusive, both genetic susceptibility and environmental factors are often associated with MS development.^5^ Emerging data indicate that gut microbiota dysbiosis and myeloid cell activation are key events in MS pathogenesis.^6^ However, detailed mechanisms of their interactions are still poorly understood.

Therapies based on myeloid regulatory cells (MRCs) have shown great potential for AD treatment. MRCs, including tolerogenic dendritic cells (DCs), M2 macrophages, and N2 neutrophils, are the most important immunoregulatory cells able to ameliorate chronic inflammatory responses.^7^ Recently, myeloid-derived suppressor cells (MDSCs) emerged as the most potent MRCs able to suppress the immune response in tumors, and the major cause of tumor resistance to immunotherapies.^8^ MDSCs include immature myeloid cells with monocyte-like morphology and phenotype labeled as mononuclear (M)-MDSCs, and immature neutrophil-like properties known as polymorphonuclear (PMN)-MDSCs. The role of MDSCs in autoimmunity has been implicated, but controversial roles for different MDSC subsets have been reported.^9^ Additionally, most preclinical studies have been exclusively based on the effects of *ex vivo* MDSCs,^10^whereas *in vitro* generated MDSCs show variable effects in the regulation of pathological immune responses.^11,12^ Therefore, the therapeutic potential of MDSCs in ADs and immunological mechanisms of MDSC-induced immune tolerance have not been sufficiently explored, primarily owing to the lack of efficient protocols for *in vitro* MDSC generation.

Granulocyte-macrophages colony-stimulating factor (GM-CSF) and interleukin (IL)-6 are critical for the induction of MDSCs during emergency myelopoiesis,^9^ and necessary for MDSC differentiation from bone marrow cells.^13^ Additionally, our previous study showed that prostaglandin (PG)E2, besides GM-CSF and IL-6, provides a critical signal for the activation of tolerogenic functions of human MDSCs,^14^ increases their resistance to pro-inflammatory stimuli, and potentiates their capacity to induce non-conventional regulatory T cell (Treg) subsets, all of which could be beneficial for MS therapy. Besides well-described immunological changes related to MS development, changes in gut microbiota composition in MS patients^6^ revealed many connections among the gut immune system, microbiota, and microbiota-derived metabolites in disease impairment.^15^ Although the association between the efficacy of immunotherapy of tumor and gut microbiota composition was established,^16^ together with our recent results on the association between gut microbiota composition and properties of *in vitro* derived anti-cancer DC therapy,^17^ similar data on MRC-based therapies of MS lacks completely. Therefore, this study aimed to investigate the potential of *in vitro*-generated MDSCs to ameliorate inflammation in an animal model of experimental autoimmune encephalomyelitis (EAE), which we showed previously as an excellent representation of RRMS.^18^ This model has two hallmarks of MS, demyelination, and the chronic relapsing disease course, and was shown to be suitable for immunological investigation, as well as testing novel immune targeting therapies. However, this model of EAE is usually characterized by spinal cord demyelination,^19^ and, in contrast to human pathology, cortical lesions, a prominent marker of chronic MS, are nearly absent. Moreover, we analyzed whether the clinical and immunological effects of MDSC application in EAE are associated with changes in the gut microbiota composition, predicted microbial metabolic pathways, and gut barrier integrity. To the best of our knowledge, this is the first report to investigate such relationships, which has enabled the identification of potential microbiota metabolites contributing to the efficacy of MDSCs in EAE.

## 2. Results

### 2.1. PGE2 potentiates suppressive functions of MDSCs generated in vitro

**Figure 1.**
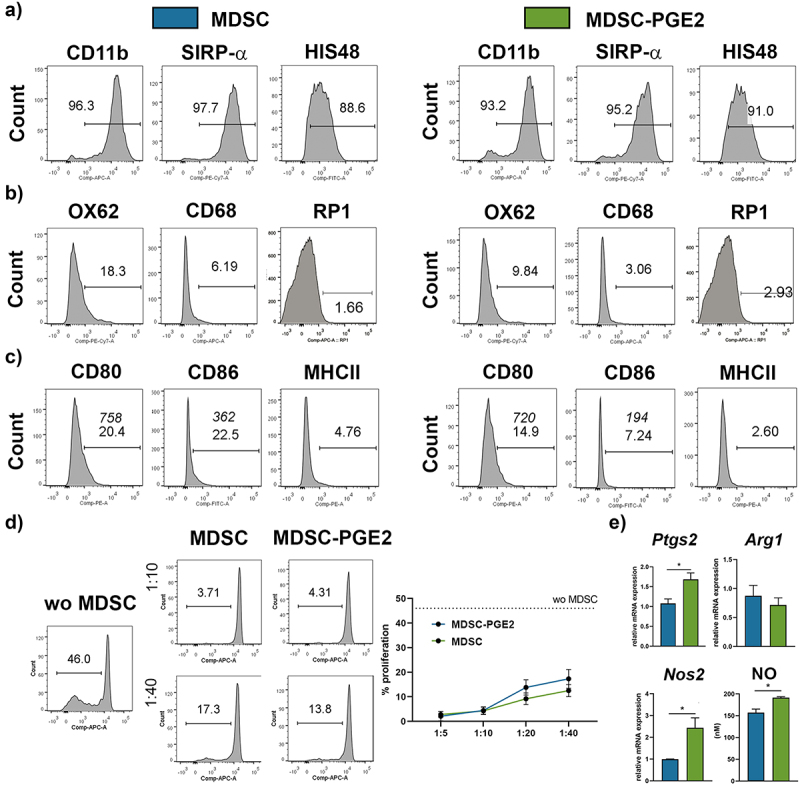
Phenotypic and functional characterisation of MDSCs. MDSCs were differentiated from bone marrow of DA rats in the presence of FLT3/GM-CSF/IL-6 (MDSCs, blue) or FLT3/GM-CSF/IL-6 and PGE2 (MDSC-PGE2, green) for 4 days. **A-C**. Representative flow cytometry histograms are showing the expression of indicated markers from one experiment, out of three with similar results (for summarised results see Supplementary Figure 1). Common markers expressed by rat MDSCs (CD11b, SIRP-α, HIS48) (**A**); myeloid lineage markers (OX62, CD68 and RP1) (**B**); functional markers (MHCII, CD86 and CD80) are shown (**C**). **D**. The proliferation of ConA-stimulated Cell Trace Far Red-labelled splenocytes (3 × 10^5^/well) is shown, in which the splenocytes were cultivated without (wo) MDSCs (dashed line) or in the presence of MDSCs or MDSC-PGE2 (each at 6 × 10^4^, 3 × 10^4^, 1.5 × 10^4^ or 0.75 × 10^4^/well, providing 1:5–1:40 MDSCs: splenocytes ratio, respectively) for 4 days. A representative analysis of 1:10 and 1:40 MDSC/splenocyte ratios is shown along with summarised data. **E**. Relative mRNA levels of COX-2, iNOS, ARG1 are shown relative to β-actin expression, as well as NO production as detected by Griess reaction method. **D-E**. The summarized data is shown as mean ± SD of three independent experiments. Student’s *t*-test was used for comparison (* *p* < 0.05).

After the differentiation of bone marrow (BM) cells in the presence of GM-CSF/FLT3/IL-6 (MDSC) or GM-CSF/FLT3/IL-6 and PGE2 (MDSC-PGE2), and enrichment with a gradient,^13,20–22^ we analyzed the phenotype of cells according to previous descriptions for rat MDSCs.^23–25^ Almost all cells expressed CD11b, SIRP-α, and HIS48, and the differentiation of cells in the presence of PGE2 did not significantly affect the expression of these markers (Figure 1A, Supplementary Figure 1). Less than 15% of the obtained cells expressed CD68 scavenger receptor,^26^ and less than 3% RP1 marker, which is usually expressed on matured granulocytes.^27^ About 20% of MDSCs expressed OX62, a marker of rat DCs, whereas this marker was less expressed on MDSCs differentiated with PGE2 (Figure 1B). In contrast to MDSCs, both CD68 and OX62 were expressed to a higher extent in BM-derived macrophages (MFs)/DCs differentiated only in the presence of GM-CSF and FLT3 (Supplementary Figure 2), according to previous protocols.^28,29^ Additionally, MF/DC preparations were predominantly composed of mononuclear cells, whereas MDSCs contained both mononuclear and polymorphonuclear cells (Supplementary Figure 2). We also analyzed the expression of markers important for the immunostimulatory function of accessory myeloid cells, that is, major histocompatibility complex (MHC) class II and co-stimulatory molecules CD86 and CD80 (Figure 1C, Supplementary Figure 1). Compared to that by BM-derived MFs/DCs, MDSCs expressed much lower levels of MHC class II, CD86, and CD80 (Supplementary Figure 2). Approximately 20% of MDSCs expressed the co-stimulatory molecules CD80 and CD86, whereas the presence of PGE2 in the cultivation medium for MDSC differentiation significantly reduced the expression of CD86 (Supplementary Figure 1). Considering that immunosuppressive potential is a prerequisite for the definition of MDSCs,^22^ we also tested the potential of the obtained myeloid cells to suppress the proliferation of concanavalin (Con)A-activated splenocytes *in vitro* (Figure 1D). MDSCs obtained with or without PGE2 strongly suppressed the proliferation of activated splenocytes, confirming that they were indeed MDSCs.

To investigate the potential mechanism of immunosuppressive activity of differentiated cells, we analyzed the expression of enzymes identified as important for MDSC suppressive functions,^23–25^ inducible nitric oxide synthase (iNOS), cyclooxygenase (COX)-2, and arginase (ARG)1 (Figure 1E). We showed that the obtained cells expressed all analyzed enzymes, and that PGE2 stimulated the expression of *Nos2* and consequently nitric oxide (NO) production, as well as *Ptgs2* mRNA expression, but not *Arg1*.

### 2.2. MDSC-PGE2 ameliorate EAE symptoms in vivo

**Figure 2.**
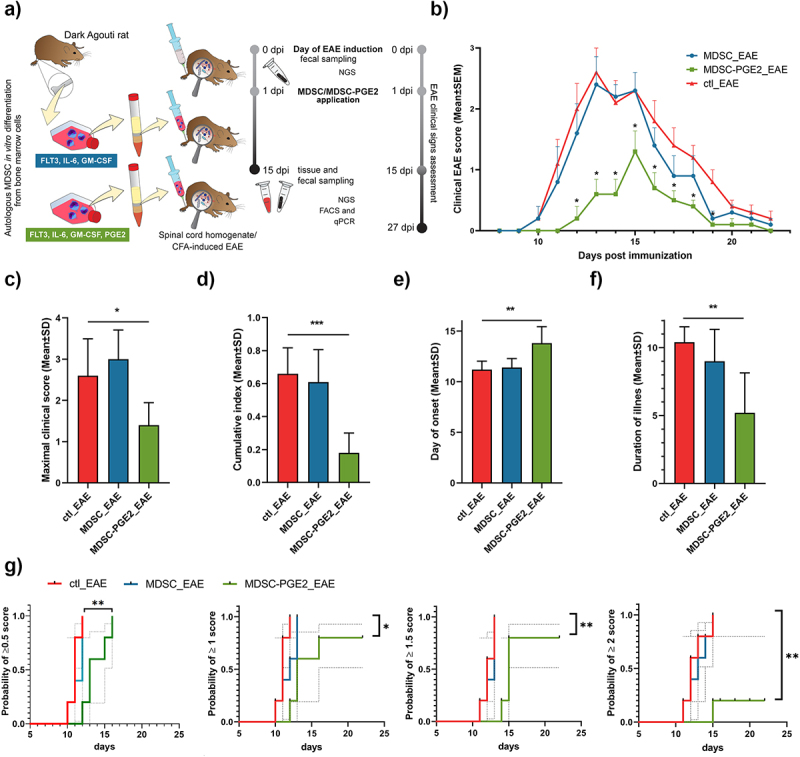
The effects of MDSC application on EAE **A**. MDSCs were differentiated from bone marrow of DA rats, either in the presence of PGE2 (MDSC-PGE2) or without PGE2 (MDSCs), as indicated. MDSCs or MDSC-PGE2 (each at 2 × 10^6^ cells/rat) were injected intraperitoneal (i.p.) as a single treatment, one day after the induction of EAE with the spinal cord tissue homogenate in complete Freund’s adjuvant (CFA) (n = 10 rats/group). The third group of rats received only PBS i.p., one day after the induction of EAE (control group). Five animals from each group were daily measured and scored for EAE signs. In each group, five animals were sampled for faecal material on the day of EAE induction (0 day per induction (dpi)). At the peak of EAE (15 dpi), faecal material, spleen, spinal cord and intestine samples were collected from these animals. **B**. The clinical score of EAE over time is shown. Each point represents mean value ± SEM of clinical scores of 5 animals in each group. Two-way ANOVA followed by Dennett’s test was used for the comparison between groups for each time point. **C-F**. Histograms depict mean values ± SD for maximal clinical score (**C**), cumulative index (**D**), day of onset (**E**) and duration of illness (**F**) for the control group (ctl_EAE – red), group that received MDSCs (MDSC_EAE – blue) and group that received MDSC-PGE2 (MDSC-PGE2_EAE – green). **(G)** Kaplan–Meier curves were used to estimate the time needed to reach clinical scores of EAE for every animal, and the log-rank test was used to compare groups. The treatments were compared with the control group using Student’s *t*-test. A representative experiment is shown, out of two with similar results. (* *p* < 0.05, ** *p* < 0.01, *** *p* < 0.001).

Considering the immunosuppressive potential of MDSCs and MDSC-PGE2 *in vitro*, we further investigated whether a similar phenomenon could be reproduced upon the transfer of these cells in an EAE model in DA rats. MDSCs and MDSC-PGE2 were administered as a single dose a day after EAE induction, followed by daily weight measurement and clinical score assessment from day 8 to day 27 (Figure 2A). Evaluation of the mean clinical score over time indicated that MDSC-PGE2-treated rats showed decreased disease impairment (Figure 2B). Maximal clinical scores, cumulative index, onset of disease symptoms, and duration of illness were used as parameters for the analysis of MDSC and MDSC-PGE2 efficacy. Our results showed that treatment with MDSC-PGE2 alone significantly decreased the maximal clinical scores in comparison to the control EAE group (Figure 2C). The cumulative index, represented as the sum of the daily mean clinical scores for a group over a given number of days, was the lowest in the EAE group treated with MDSC-PGE2 and significantly differed from that in the control EAE group (Figure 2D). The results also showed that MDSC-PGE2 significantly delayed the onset of EAE symptoms compared to the control EAE group (Figure 2E). Furthermore, only the treatment of animals with MDSC-PGE2 significantly shortened the duration of disease compared to the control EAE group (Figure 2F). Although these parameters were significantly improved by MDSC-PGE2 treatment after EAE induction, all animals developed some degree of disease, with at least partial tail weakness (score 0.5) (Figure 2G). By analyzing the incidence of symptoms per day of the disease, we observed that all animals in the control EAE and MDSC groups, but only one animal in the MDSC-PGE2 group, developed a clinical score ≥2. Altogether, these results demonstrated that only MDSC-PGE2 significantly ameliorated EAE symptoms *in vivo*.

### 2.3. MDSC-PGE2 suppressed spinal cord infiltration and systemic inflammation in EAE

IL-17-secreting CD4^+^ Th17 and interferon (IFN)-γ secreting cells passing the BBB have been recognized as the most important pathogenic cells in neuroinflammation.^30^ To investigate how MDSC and MDSC-PGE2 application affected the local immune response in EAE animals, we collected and analyzed the spinal cords and infiltrating cells. Additionally, to assess the effects of MDSCs on systemic immune responses, we analyzed the spleen of each animal. At the peak of the disease (15^th^ day post-immunization), animals in the EAE group displayed multifocal infiltrates in the white matter of spinal cord cross-sections made at the lumbar region (Supplementary Figure 3), and a similar histological image was observed in the group treated with control MDSCs. In contrast, much smaller infiltrates or no infiltrates were found in animals treated with MDSC-PGE2 after EAE induction. Additionally, epifluorescence analysis of CD45/myelin basic protein (MBP) co-stained cross-sections suggested that the expression of MBP in the white matter of EAE and MDSC groups was much lower than in the MDSC-PGE2 group (Supplementary Figure 3).

**Figure 3.**
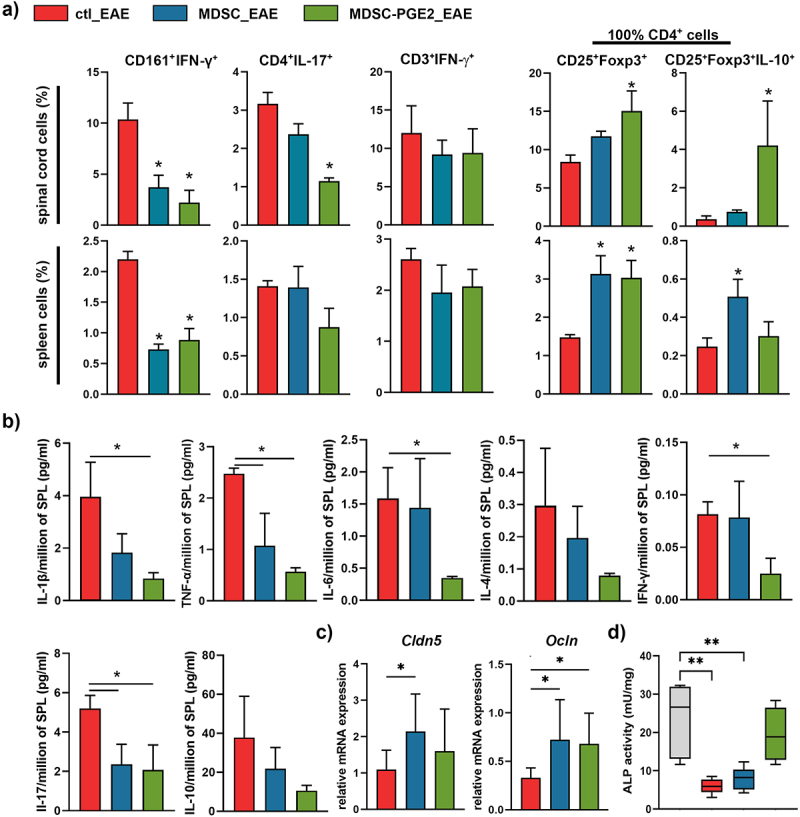
Immune response in animals treated with MDSCs and MDSC-PGE2 at the peak of EAE. **A**. The percentages of interferon (IFN)-γ-producing NK (CD161^+^IFN-γ^+^), Th17 (CD4^+^IL-17^+^) and (IFN)-γ-producing T (CD3^+^IFN-γ^+^) lymphocytes in the spleen and spinal cord samples are shown, after their analysis upon isolation from animals (n = 5 animals in each group) at the peak of EAE (15dpi) in the control (ctl_EAE, red) group, and groups treated with either MDSCs (MDSC_EAE, blue) or MDSC-PGE2 (MDSC-PGE2_EAE, green). Data was obtained by flow cytometry analysis as shown in Supplementary Figure 4. The percentages of regulatory T cells (Tregs) (CD25^+^FoxP3^+^) and IL-10-producing Tregs (CD25^+^FoxP3^+^IL-10^+^) were shown from the total gated CD4^+^ lymphocytes (the gating strategy is shown in Supplementary Figure 5). The results on total cell numbers are shown in Supplementary Figure 6. **B**. The levels of cytokines (interleukin (IL)-1β, tumor necrosis factor (TNF)-α, IL-6, IL-4, IL-17, IFN-γ and IL-10) were measured from *ex vivo* splenocytes isolated from each animal (as in **A**), and cultivated (2 × 10^6^/mL/well of 24-wells plate) for 24 h in the complete RPMI medium in the presence of ConA. **C**. The relative mRNA levels of claudin and occludin in intestinal samples of animals treated as in **(A**), were calculated relative to β-actin and presented as fold change expression relative to healthy animals **D**. The activity of alkaline phosphatase in faecal material collected at the peak of EAE, as well as from healthy animals were analysed as a measure of intestinal barrier integrity. **A-D**. Data is shown as mean ± SD (n = 5) from one experiment, out of two with similar results. One-way ANOVA followed by Tukey *post hoc* test was used for comparison (* *p* < 0.05, ** *p* < 0.01) to control EAE (ctl_EAE).

To further characterize cellular infiltrates in the spinal cord, we used flow cytometry analysis (Figure 3A, Supplementary Figures 4 and 5), and the total number of cells of interest (Supplementary Figure 6) was calculated according to the live cell count using Trypan blue after the isolation of cells from the tissues. The analysis suggested that the application of MDSC-PGE2 after EAE induction significantly lowered the percentage and total number of CD4^+^IL-17^+^ cells in the spinal cord but not spleen. In addition, both MDSCs and MDSC-PGE2 strongly reduced the percentage and total number of IFN-γ^+^ CD161^+^ NK cells, both in the spinal cord and spleen. In contrast, the percentage of IFN-γ-producing T cells in the CNS and spleen did not differ significantly between the groups (Figure 3A, Supplementary Figure 6).

Next, we analyzed how treatment of animals with MDSCs after EAE induction affected regulatory T cell populations. We found a higher relative abundance of CD4^+^ CD25^+^Foxp3^+^ cells in the spinal cord of animals treated with MDSC-PGE2, and in the spleen of animals treated with either MDSCs or MDSC-PGE2. Furthermore, an increase in CD25^+^Foxp3^+^IL-10^+^ expression in CD4^+^ cells and the total number of these cells was observed in the spinal cord of animals treated with MDSC-PGE2, whereas a significant increase in these cells was only detected in the spleen of the MDSC-treated group (Figure 3A, Supplementary Figure 6).

To better understand the potential mechanisms of MDSC and MDSC-PGE2 immunosuppressive activity in EAE, we additionally measured IL-1β, tumor necrosis factor (TNF)-α, IL-6, IL-4, IL-17, IFN-γ, and IL-10 production by splenocytes isolated from the animals at the peak of the disease (Figure 3B). The results indicated a significant reduction in pro-inflammatory cytokine (IL-1β, TNF-α, IL-6, IFN-γ, and IL-17) production by *ex vivo* splenocytes from the group treated with MDSC-PGE2, whereas splenocytes isolated from MDSC-treated animals showed only decreased levels of TNF-α and IL-17.

### 2.4. MDSC-PGE2 migrated into gut lymphoid tissues and prevented EAE-induced changes in the intestinal barrier markers

In addition to systemic and localized inflammation, it is known that the inflammation induced in EAE can lead to intestinal barrier impairment and translocation of bacterial antigens into the bloodstream that elicit immune response further.^31^ To analyze the effects of applied treatments on the intestinal barrier of immunized rats, we first analyzed the expression of tight junction proteins, Claudin and Occludin, which are critical for the maintenance of barrier integrity. These analyses showed that both MDSC and MDSC-PGE2 application increased the mRNA expression of tight junction proteins in the intestine of EAE-induced animals (Figure 3C), indicating the protective effect of these treatments on intestinal barrier integrity. In addition to changes in the expression of mRNA for tight junction proteins, the activity of fecal alkaline phosphatase (ALP) as a measure of intestinal barrier integrity,^32^ was decreased in the EAE group as well as in the MDSC-treated group, but remained high in the feces of the MDSC-PGE2-treated group (Figure 3D).

Considering the strong effect of MDSC-PGE2 on the amelioration of EAE symptoms and preservation of ALP activity, we next monitored the migration pattern of carboxyfluorescein succinimidyl ester (CFSE)-labeled MDSC-PGE2 in animals in which EAE was induced and in the control non-immunized animals, followed by the collection of lymphoid tissues and spinal cords on the 3^rd^, 7^th^, and 10^th^ days post-administration (Supplementary Figure 7). On the 3^rd^ day, the highest percentage of CFSE-labeled cells in both EAE and non-immunized animals was found within C45^+^CD11b^+^ cells in Peyer’s patches and mesenteric lymph nodes, whereas a smaller percentage was detected in inguinal lymph nodes (Supplementary Figure 7). On the 7^th^ day, CFSE-labeled myeloid cells were found in Peyer’s patches, lymph nodes, and the spinal cord of animals with EAE, and their presence was still detectable in the spinal cords and Peyer’s patches on the 10^th^ day of analysis. However, in the control non-immunized animals, most CFSE-labeled myeloid cells were found in the spleen on days 7 and 10. Non-immunised control animals did not show significant levels of CD45^+^ cells within the spinal cord; therefore, we could not detect any migration of CFSE-labeled cells in the spinal cord (data not shown). These results suggest that the gut immune system is the early site of MDSC-PGE2 migration in EAE, preceding the migration to the spinal cord where they could regulate the disease.

### 2.4. MDSCs prevented EAE-induced reduction of microbial diversity

Gut microbiota imbalance could be associated with failure of immune self-tolerance leading to autoimmunity^33^ or contributing to an already established inflammation.^34^ There is already growing evidence pointing to altered intestinal microbiota composition being an important environmental factor in different AD pathogenesis.^6^ Also, the strong relationship between the effectiveness of anti-tumor therapy and gut microbiota composition has been described previously,^16^ highlighting the importance of investigating the gut microbiota properties in the context of immune-modulation-based therapies.

**Figure 4.**
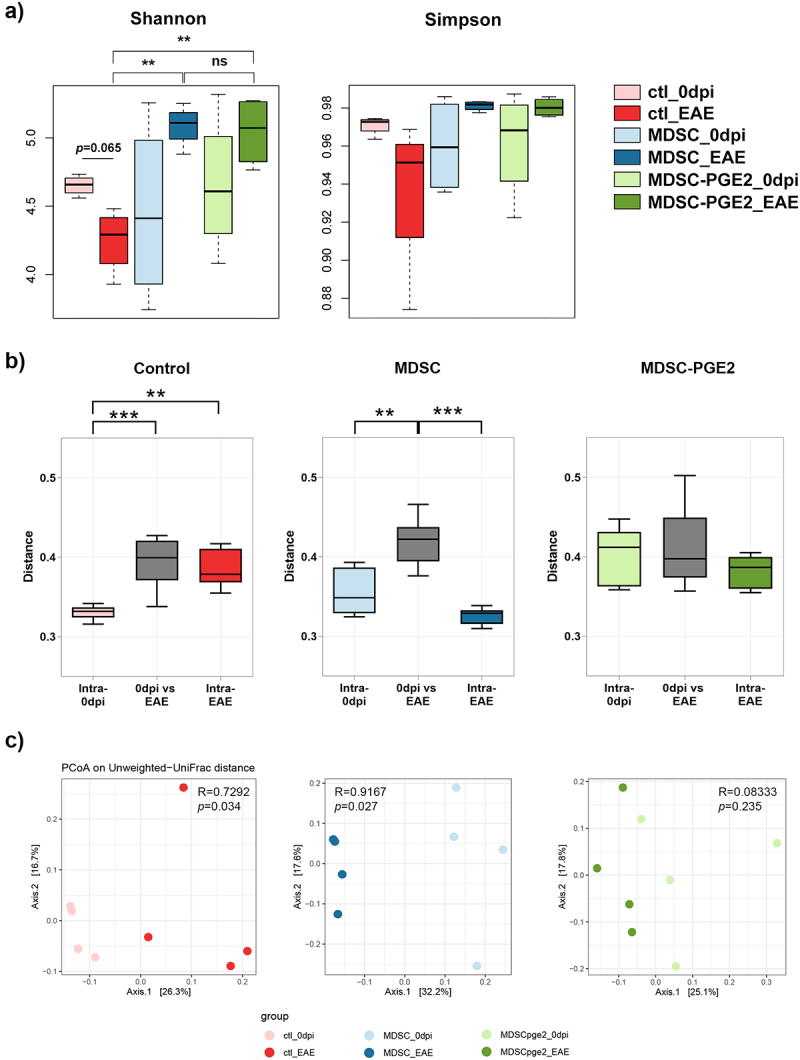
Diversity of intestinal microbiota in control, MDSCs and MDSC-PGE2-treated animals with EAE. **A**. Boxplots of Shannon and Simpson indices (α-diversity) are shown within the control EAE group (at 0dpi – ctl_0dpi, light red and at 15dpi – ctl_EAE, dark red), MDSC treated EAE group (at 0dpi – MDSC_0dpi, light blue and at 15dpi – MDSC_EAE, dark blue) and MDSC-PGE2 treated EAE group (at 0dpi – MDSC-PGE2_0dpi, light green and at 15dpi – MDSC-PGE2_EAE, dark green). Statistical significance between the matched observations (0dpi vs 15dpi) was tested using Welch Two Sample *t*-test, and the unmatched comparisons were tested using unpaired Welch Two Sample *t*-test, both followed by Benjamini-Hochberg procedure for controlling the false discovery rate. **B-C**. Microbial beta diversity was compared using boxplots **(B)** and principal coordinates analysis (PCoA) plots **(C)** based on Unweighted UniFrac distances. For the boxplots, Intra-0dpi (light color) indicate distances of gut microbiota composition among the animals in one group before the immunization, Intra-EAE (darker color) indicate distances of gut microbiota composition among the animals in one group at the time of EAE peak, while 0dpi vs EAE (gray for all three groups) indicates distances of gut microbiota compositions between the samples collected before immunization and at the peak of the disease. The Unpaired Welch Two Sample *t*-test was used to determine the statistical significance between variables. For the PCoA percent variance explained by each principal coordinate is denoted on the axes. Statistical analysis was performed by ANOSIM. (** *p* < 0.01, *** *p* < 0.001).

Considering the migration pattern of MDSCs after application in EAE, the effects of these treatments on intestinal tight junction proteins, and the level of fecal ALP activity, indicating the protection of intestinal barrier integrity in the MDSC-PGE2 group, we wondered whether there was a relationship between gut microbiota diversity and the effects of MDSCs and MDSC-PGE2 in EAE. Therefore, we first calculated alpha diversity metrics (Shannon and Simpson indices) and compared them using paired Welch’s two-sample *t*-test for comparison of microbiota diversity before immunization, and at the time of EAE peak by using the unpaired Welch’s two-sample *t*-test for comparison between the groups. The induction of EAE in the control group of animals resulted in a decrease in microbiota diversity compared to the time point before immunization, whereas MDSC as well as MDSC-PGE2 administration preserved the microbial diversity in immunized animals (Figure 4A). We also examined the differences in the alpha diversity indices between the three groups at the peak of the disease, which showed statistical differences in the Shannon index between the control and MDSC-treated groups and between the control and MDSC-PGE2 groups. Alpha diversity analysis revealed no difference between the MDSC and MDSC-PGE2 groups.

Next, to examine the beta diversity in the control, MDSC, and MDSC-PGE2 groups, we generated a matrix of pairwise distances between the samples using Unweighted UniFrac algorithm, which considers taxa presence-absence information (Figure 4B). Intra-0dpi indicates distances of gut microbiota composition between the animals in one group before immunization, intra-EAE indicates distances of gut microbiota composition between animals in one group at the time of EAE peak, whereas 0dpi vs EAE indicates distances of gut microbiota compositions between the samples collected before immunization and at the peak of the disease. The results showed that EAE induction resulted in higher distances between the gut microbiota composition of animals at the time of the EAE peak (ctl group, intra-EAE). Similar distances were observed in the control group between the samples collected before immunization and at the time of the EAE peak (ctl group, 0 dpi vs. EAE). In the MDSC group, significantly higher distances were observed between the gut microbiota composition of animal samples collected before immunization and at the time of EAE, compared to distances within these two groups. There were no statistically significant differences in beta diversity in MDSC-PGE2 treated group of animals. Similar results were observed when principal coordinate analysis (PCoA) was performed on the same distance matrix (Figure 4C). Analysis of similarities (ANOSIM) showed significant similarities within the group community composition when comparing the animals before immunization and at the EAE peak in the control EAE group (R = 0.7292, *p* = 0.034), indicating significant inter-group variability. Similar results were observed in the MDSC group (R = 0.9167, *p* = 0.027). In contrast, according to this analysis (R = 0.08333, *p* = 0.235), there were no significant differences in the microbiota composition in MDSC-PGE2 group between samples collected before EAE induction and at the peak of the disease.

### 2.5. MDSC-PGE2 prevented large alternation in microbiota composition induced by EAE

**Figure 5.**
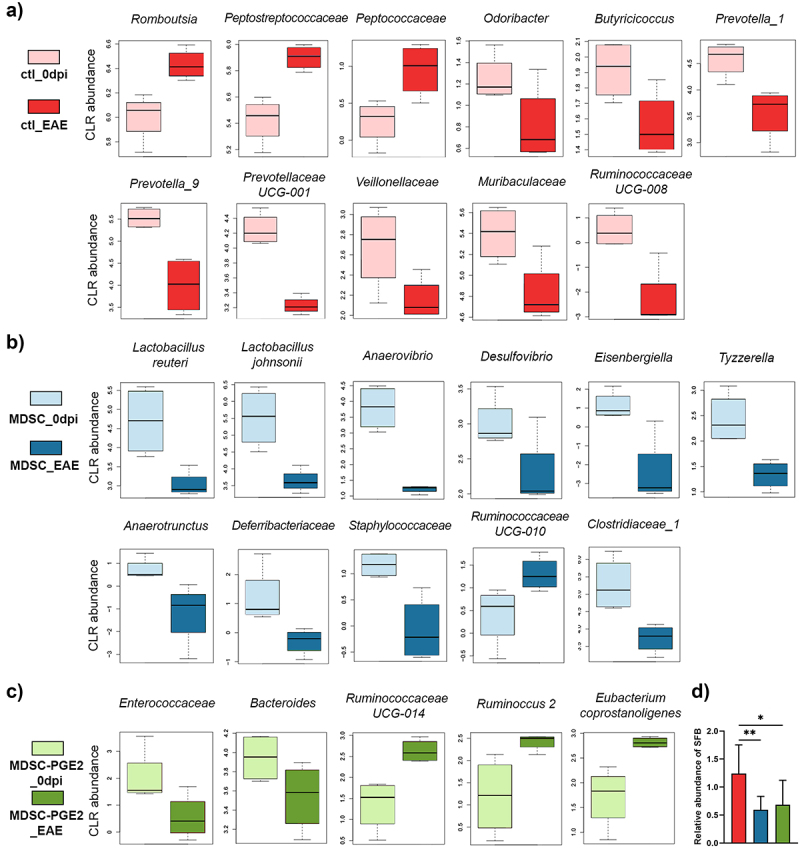
Alternation in microbiota composition induced in control, MDSC- and MDSC-PGE2-treated animals with EAE. Boxplots present the taxa-centered log ratio (CLR)-transformed abundances of taxa before the immunization/treatment application (0dpi, light colors) and at the peak of EAE (EAE, darker colors) in control animals (**A**) (red), animals treated with MDSCs (**B**) (blue) and animals treated with MDSC-PGE2 (**C**) (green). Taxa with ANCOM W > 1 were additionally tested for statistical significance using Pairwise Wilcoxon test or paired *t*-test followed by Benjamini-Hochberg p-value correction (only statistical significant comparisons are shown). **D**. qPCR analysis of faecal DNA of the control, MDSC- and MDSC-PGE2-treated animals with EAE, targeting segmented filamentous bacteria (SFB) are presented on the histograms. One-Way ANOVA/Tukey *post hoc* test was used for comparisons (* *p* < 0.05, ** *p* < 0.01).

Previous data showed that MS as well as EAE are associated with changes in gut microbiota, either in enriched prevalence of harmful bacteria or depletion in beneficial bacteria.^6,15^ To identify the changes in gut microbiota composition resulting from EAE induction, as well as the effects of MDSC and MDSC-PGE2 administration, we analysed the differences between the samples collected before immunization and at the time of EAE peak at the genus, family, and species levels. On the one hand, compared with the microbiota composition before the induction of EAE, the relative abundance of the genus *Romboutsia*, as well as two families, *Peptostreptococcaceae* and *Peptococcaceae*, increased at the time of EAE peak in the control EAE group. On the other hand, reduction in *Odoribacter, Butyricicoccus*, and genera from the *Prevotellaceae* family (*Prevotella_1, Prevotella_9*, and *Prevotellaceae UCG-001*), but also in *Veillonellaceae, Muribaculaceae*, and *Ruminococcaceae UCG-008* were observed at the time of EAE peak in the control group (Figure 5A).

In the group treated with MDSCs (Figure 5B), we observed lower abundances of *Lactobacillus reuteri* and *Lactobacillus johnsonii* at the time of EAE peak, as compared to the gut microbiota composition before immunization. In addition, the relative levels of *Anaerovibrio, Desulfovibrio, Eisenbergiella, Tyzzarella*, and *Anaerotrunctus* were reduced at the same time. In addition to families encompassing these genera/species, families *Defferibacteriaceae, Staphylococcaceae, Ruminococcaceae UCG-010* and *Clostridiaceae_1* were reduced in the MDSC treated group.

Interestingly, a minimal alteration in microbiota composition was observed in rats treated with MDSC-PGE2 at the time of EAE peak, and the only reductions observed were in the family *Enterococcaceae* and genus *Bacteroides*, together with enrichment of family *Ruminococcaceae UCG-014*, genus *Ruminococcus* 2, and species *Eubacterium coprostanoligenes* (Figure 5C).

Finally, the presence of segmented filamentous bacteria (SFB), gut commensals found in different species, including humans and rodents^35^ were investigated using quantitative polymerase-chain reaction (qPCR). Enrichment in these bacteria has been associated with MS severity.^36^ We performed relative quantification of specific amplicons amplified from isolated fecal DNA and observed a lower abundance of SFB in MDSC and MDSC-PGE2 treated rats compared to control rats at the peak of EAE (Figure 5D). These data suggested that EAE significantly affected the composition of gut microbiota in animals. Disturbance in gut microbiota composition was also detected in animals treated with MDSCs, but only a few changes were detected in animals treated with MDSC-PGE2.

### 2.6. MDSC-PGE2 favored tolerogenic microbial metabolic pathways in gut microbiota

**Figure 6.**
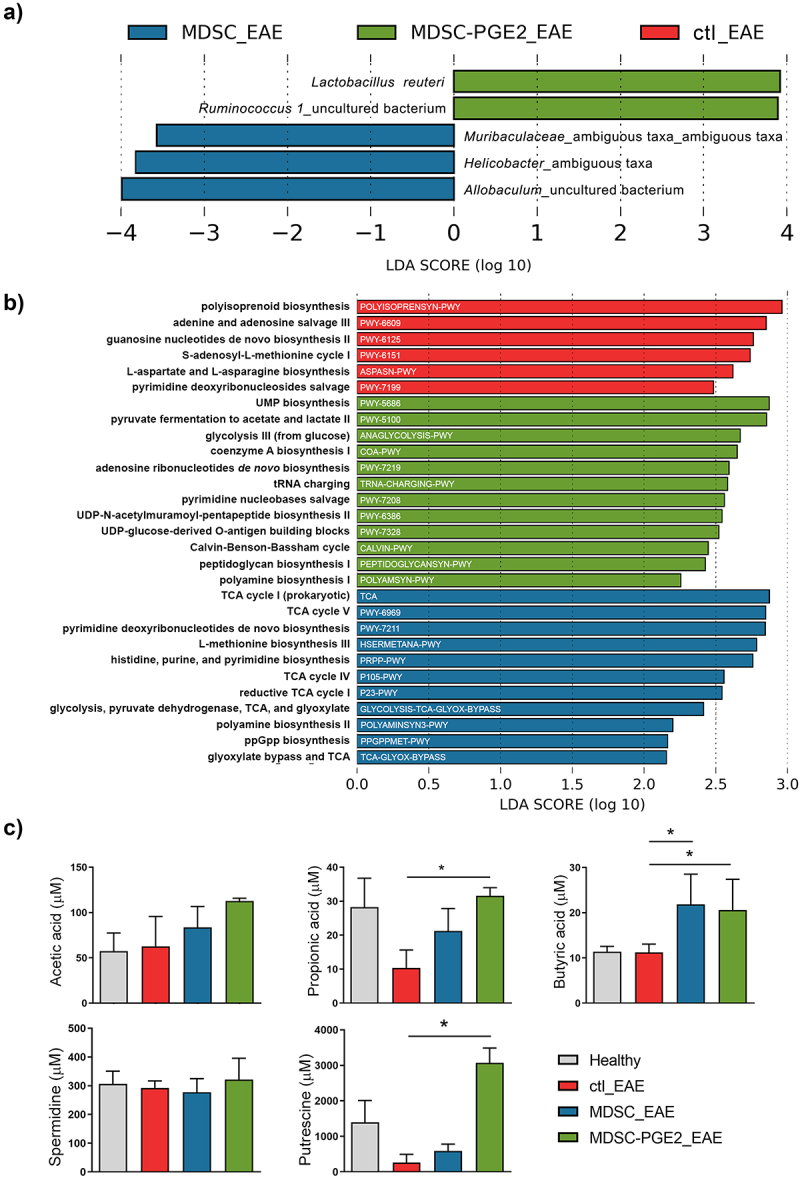
Microbial signature identification across the groups at the peak of EAE. **A**. Linear discriminant analysis (LDA) effect size (LEfSe) on the species level, comparing all three groups at once (control group at the peak of EAE, ctl_EAE in red; group treated with MDSC at the peak of EAE, MDSC_EAE in blue; group treated with MDSC-PGE2 at the peak of EAE, MDSC-PGE2_EAE in green) are shown. Histogram shows only the most differentially abundant features among the groups passing logarithmic LDA score above 3.5 along with alpha value of <0.05 for factorial Kruskal-Wallis and all-against-all strategy for multi-class analysis. Species with positive LDA scores (green bars) are more prominent in the group treated with MDSC-PGE2, whereas negative LDA scores (blue bars) indicate species enriched in the group treated with MDSC. **B**. Differentially abundant predicted metabolic pathways generated using PICRUSt2 pipeline with default options, unveil discriminative predicted pathways among the groups at the peak of EAE. Histogram depicts pathways meeting an LDA significant threshold of >2 in the LEfSe Galaxy framework. MetaCyc metabolic pathways ID are shown as white text on bars, while pathways’ full names are shown on the y-axis. Color legend is the same as described above. **C**. The levels of SCFAs and polyamines were measured using High-performance liquid chromatography (HPLC) in faecal samples, collected from the animals at the peak of the disease, as well as from healthy animals. The level of SCFAs and polyamines were compared between the groups by Kruskal–Wallis test (* p < 0.05).

In addition to comparing the changes in gut microbiota within the treatment groups before immunization and at the time of EAE peak, we further explored the differences between the groups at the time of EAE peak (Figure 6A). To compare the relative abundances between the groups at the time of the EAE peak, we conducted a linear discriminant analysis effect size (LEfSe) pipeline using Galaxy module, with a logarithmic LDA score above 3.5 and other default parameters. *Lactobacillus reuteri* and *Ruminococcus 1* were shown to be microbial markers of the MDSC-PGE2 group, whereas *Muribaculaceae, Helicobacter*, and *Allobaculum* were enriched in the MDSC group at the EAE peak. Notably, no microbial markers were detected in the control group at the time of the EAE peak.

Gut microbiota produce soluble metabolites that reach the bloodstream by passing through the intestinal barrier.^37^ To investigate potential microbial functional composition, we performed phylogenetic investigation of communities by reconstruction of unobserved states (PICRUSt)2 analysis followed by LEfSe analysis to determine the differences in predicted microbial metabolic functions between the groups, showing only pathways meeting an LDA significance threshold of >2 (Figure 6B). These analyses showed several pathways involved in the biosynthesis of purine (PWY-6125) and pyrimidine (PWY-7199) nucleotides, amino acids (ASPASN-PWY), S-adenosyl-L-methionine cycle (PWY-6151), and polyprenyls (POLYISOPRENSYN-PWY), and one pathway in charge of purine nucleotide salvage (PWY-6609), as potential markers of the control group at the EAE peak.

The MDSC group was characterized by the highest number of enriched pathways involved in the biosynthesis of purine (PWY-7219) and pyrimidine (PWY-5686, PWY-7208) nucleotides, coenzyme A (COA-PWY), aminoacyl-tRNA (TRNA-CHARGING-PWY), UDP-N-acetylmuramoyl-pentapeptide (PWY-6386), peptidoglycan (PEPTIDOGLYCANSYN-PWY), lipopolysaccharide (PWY-7328), and polyamines (POLYAMSYN-PWY), fermentation to short-chain fatty acids (SCFAs) (PWY-5100), CO_2_ fixation (CALVIN-PWY), and generation of precursor metabolites and energy (ANAGLYCOLYSIS-PWY).

The analyses showed that the MDSC-PGE2 group was marked by enrichment of pathways involved in the biosynthesis of purine (PRPP-PWY) and pyrimidine nucleotides (PWY-7211, PRPP-PWY), amino acids (HSERMETANA-PWY), metabolic regulators (PPGPPMET-PWY), polyamines (POLYAMINSYN3-PWY), glycolysis (TCA-GLYOX-BYPASS), CO_2_ fixation (P23-PWY), and precursor metabolites and energy generation (TCA, PWY-6969, P105-PWY, GLYCOLYSIS-TCA-GLYOX-BYPASS).

To evaluate whether the changes observed in microbial metabolic pathway prediction corresponded to the actual changes in the levels of key microbial metabolites in animal feces, we measured the concentrations of SFCAs (butyric, propionic, and acetic acid) and polyamines (spermidine and putrescine) in fecal samples from the three groups at the EAE peak, as well as in healthy, non-treated animals. The results suggest that the fecal levels of acetic acid and spermidine did not differ significantly between the groups of tested animals. The levels of propionic acid and putrescine were lowered at the peak of disease in control (EAE) group, as compared to their levels in the healthy animals. This reduction was lower in the MDSC-treated group, particularly in the MDSC-PGE2-treated group, in which the levels of putrescine exceeded those of healthy control animals. Interestingly, the levels of fecal butyric acid did not change significantly in the EAE peak as compared to healthy animals, but were significantly increased in the groups of animals treated with MDSCs and MDSC-PGE2 (Figure 6C). These results suggest that MDSCs, especially MDSC-PGE2 treatment after EAE induction, can recover or even increase the levels of metabolites involved in the immunoregulatory mechanisms by the gut microbiota, as predicted by the PICRUSt2 analysis followed by LEfSe analysis.

## 3. Discussion

MDSCs are a heterogeneous population of myeloid cells with strong immunosuppressive properties.^8^ Although the role of MDSCs in cancer has been widely investigated, much less is known about their potential to modulate ADs and whether they display any therapeutic potential. The effects of MDSCs on the course of EAE have been studied previously, predominantly in adoptive transfer experiments of *ex vivo* isolated MDSCs in mice,^10^ whereas similar reports on *in vitro* generated MDSCs are quite scarce. In the present study, we prepared MDSCs from rat BM cells using FLT3, GM-CSF, and IL-6, as these factors were shown to be necessary for the induction of STAT-3 and suppressive properties in MDSCs.^9,13,20–22^ Without IL-6, FLT3, and GM-CSF induces immature DCs and MFs, expressing high levels of OX62, CD68, CD86, CD80, and MHC class II,^28,29^ which was confirmed in this study as well. We have previously shown that PGE2 is a potent activator of suppressive properties in human M-MDSCs and their capacity to induce Tregs.^14^ Considering that PGE2 is a major COX-2 product that can enhance MDSC accumulation and suppressive activity,^14,38^ we additionally supplemented the media for MDSC differentiation with PGE2 to obtain MDSC-PGE2. Both mononuclear and polymorphonuclear cells were present in the low-density fraction upon differentiation of MDSCs, and these cells displayed CD11b^+^SIRPa^+^His48^+^ MHC class^−^ phenotype, which corresponds to the previously described phenotype of rat MDSCs.^23^ Although OX62, usually used as a marker of rat DCs, was expressed on BM-derived MDSCs, the total expression of MHC class II molecules (OX6) was much lower than OX62 expression, suggesting that the total percentage of DCs (OX62^+^OX6^+^) in our MDSC population was quite low. Unfortunately, we could neither confirm nor exclude the contribution of contaminant myeloid cells to the total effect observed *in vitro* and *in vivo*, as such tools for rat MDSCs are still lacking.

Analysis of molecules related to the inflammatory functions of myeloid cells showed that the obtained MDSCs expressed very low levels of CD80/CD86 co-stimulatory molecules, and the expression of these molecules in cells differentiated in the presence of PGE2 was negligible. Furthermore, we confirmed the expression of COX-2, iNOS, and ARG1 in the obtained myeloid cells. PGE2 stimulated the expression of COX-2 and iNOS but did not significantly change the expression of ARG1. Although increased expression of COX-2 in human tumor tissue is followed by higher PGE2 production resulting in ARG1 induction in MDSCs,^39^ the lack of stimulation of ARG1 expression in our setting could be a characteristic of MDSCs in rats. This is in accordance with the results published by Jia et al.^24^ showing that glioma-infiltrating MDSCs expressed all three enzymes, but NO production was the primary mechanism of suppression. In addition, Dugast et al.^25^ showed that rat MDSCs preferentially suppress effector T cells in a contact-dependent and iNOS-dependent manner. Our results indicated that MDSCs generated *in vitro* can suppress Con-A-induced proliferation of splenocytes when differentiated with PGE2 or without them. However, only MDSCs differentiated in the presence of PGE2 ameliorated EAE symptoms *in vivo*, delayed the onset of the disease, and shortened the illness duration compared to the control EAE group. Th1 and Th17 cells infiltrating the CNS primarily contribute to the pathogenesis of MS and EAE, whereas Tregs limit inflammation.^3,40^ Our results indicate that MDSC application did not affect the IFN-γ-producing T cell population. In contrast, both MDSCs and MDSC-PGE2 reduced the percentage of NK-producing IFN-γ cells (CD161^+^IFN-γ^+^) in the spinal cord and displayed a similar suppressive effect on IFN-γ-producing NK cells in the spleen. Both inflammatory and immunoregulatory roles of IFN-γ producing NK cells have been proposed in EAE.^41^ Some studies showed that NK cells are the main source of IFN-γ production in the initiation stage of EAE, which is probably important for the migration of encephalitogenic T cells into the CNS.^42^ In the later phases, IFN-γ-producing NK cells were shown protective in EAE via inhibition of Th17 cell differentiation.^43^ In light of these results, it could be hypothesized that MDSCs affected the function of NK cells early at the time of the immunization/treatment application, which could be one of the factors contributing to weaker symptoms and slower development of EAE in the MDSC-PGE2 group. Consistent with the protective effects of MDSC-PGE2, only this treatment reduced the percentage of Th17 (CD4^+^IL-17^+^) cells in the spinal cord. In addition, we showed significantly reduced production of pro-inflammatory cytokines (IL-1β, TNF-α, IL-6, IFN-γ, and IL-17) in splenocytes isolated at the peak of EAE from animals treated with MDSC-PGE2, whereas only TNF-α and IL-17 were reduced in splenocytes isolated from MDSC-treated animals. Along with these results, both types of MDSCs potentiated the induction of Treg (CD4^+^CD25^+^Foxp3^+^) cells at the systemic level; however, treatment with MDSC-PGE2 increased the percentage of Treg cells in the spinal cord and their capacity to produce IL-10. Potential mechanisms for inducing IL-10-producing Tregs and downregulation of Th17 cells, but not IFN-γ-producing T cells, could be explained by the increased capacity of MDSC-PGE2 to produce NO. Namely, Neidbala et al.^44^ showed that NO-induced Tregs reduce Th17 cells but not Th1 cells, in an IL-10-dependent manner, which is in line with our findings. Additionally, we showed that human M-MDSCs induced by PGE2 display an increased capacity to induce IL-10-producing Tregs, predominantly via ILT3 and ILT4.^14^ However, the role of ILT3 and ILT4 in rat MDSC-mediated suppression of EAE is still unknown. Therefore, additional investigations are needed to delineate the molecular mechanisms involved in the induction of Tregs by MDSC-PGE2 *in vivo* and the subsequent amelioration of EAE by these cells.

Many studies have reported that changes in the gut microbiota could play a role in MS development and disease severity in patients.^6,15^ The fact that encephalitogenic Th17 cells are primed in the gut before the symptoms of EAE appear,^45^ and induce gut barrier damage and inflammation,^46^ indicates that the gut immune system is critical for the regulation of EAE. Here, we showed that MDSC-PGE2 migrates into the gut immune system compartments (Payer’s patches and MLN) as early as 3 days post-injection and remain there afterwards. In addition, NO was shown to protect the gut barrier function in certain circumstances,^47^ and a previous report suggested that MDSCs expressing NOS2 accumulate in the gut to suppress inflammation in a T cell-dependent murine colitis model.^48^ Therefore, it is tempting to speculate that the early migration of NO-producing MDSC-PGE2 in the gut could be responsible for the observed suppression of Th17 cells at the systemic level and their lower infiltration into the CNS. Further studies are necessary to confirm this hypothesis.

To the best of our knowledge, this is the first report to show that the application of MDSCs generated *in vitro* in EAE can prevent the disturbance of gut microbiota composition and gut barrier functions. Microbiota–host interactions are based on soluble microbiota-derived metabolites, such as SCFAs, tryptophan derivatives, modified bile acids, and polyamines,^49^ with different effects on host immune function. Here, we have found that EAE is associated with a reduction of different potentially SCFA-producing bacteria,^50^ such as family *Prevotellaceae* (*Prevotella_1, Prevotella_9*, and *Prevotellaceae UCG-001), Odoribacter, Butyricicoccus*, and *Ruminococcaceae UCG-008*, as well as *Muribaculaceae* previously associated with propionate production. The reduction in relative abundances of *Odoribacter* and *Butyricicoccus*^50^ as well as *Prevotella*^6^ and *Veillonellaceae*^15^ have been previously associated with more severe symptoms in MS. In addition to the reduction of these protective bacteria, EAE induction in our model was associated with the overgrowth of the genus *Romboutsia*, families *Peptostreptococcaceae* and *Peptococcaceae*, all previously associated with MS and EAE.^51–53^

Interestingly, the high number of changes in relative abundances at different taxonomic levels at the time of the EAE peak were shown to be associated with MDSC application. A reduction in potentially harmful bacteria was observed in control MDSC-treated animals at the time of the EAE peak. Thus, we observed a lower relative abundance of *Desulfovibrio*, known for sulfate-reducing properties and hydrogen sulfide production in the gut, which is potentially associated with ulcerous colitis induction.^54^ Also, a lower relative abundance of *Eisenbergiella* and *Tyzzerella* (both from *Lachnospiraceae* family), which are considered pro-inflammatory bacteria,^51,52^ was observed at the EAE peak in the MDSC-treated group. The reduction in *Anaerotruncus* was positively associated with IL-17 production in an infection model,^53^ *Deferribacteriaceae* family, involved in the recruitment of inflammatory cells into the CNS in MS and EAE,^55^ and *Staphylococcaceae*, strongly associated with inflammatory diseases,^56^ were observed at the time of EAE peak in the MDSC-treated group. Also, some of the beneficial bacteria were increased in animals treated with MDSCs, such as SCFA-producing *Ruminococcaceae UCG-010*.^57^ In addition, the genera *Allobaculum* and *Helicobacter* observed by LEfSe analysis as discriminatory microbial markers of MDSC-treated group were previously described as mucin degraders, negatively correlated with inflammation mediators in mice,^58^ and to provide protection against inflammatory demyelination in the CNS,^47^ respectively. In contrast, some bacteria with already characterized anti-inflammatory properties decreased in this group. These included species of the genus *Lactobacilli, Lb. reuteri*,^59^ and *Lb. johnsonii*,^60^ genus *Anaerovibrio*, some of which were shown to be associated with immunoregulatory metabolite production, such as propionate and acetate^61^ and polyamines (putrescine, cadaverine, and tyramine)^62^ and *Clostridiaceae*_*1*, whose members were shown to be involved in bile acid metabolism.^63^ Therefore, it could be concluded that the extensive disturbance of gut microbiota in the MDSC-treated group might have prevented the efficacy of this treatment.

In contrast to broad changes in the control and MDSC-treated animals, the relative abundances of just a few bacterial taxa changed at the time of the EAE peak in animals treated with MDSC-PGE2. Among them, the genera *Enterococcus* and *Bacteroides*, previously associated with colitis induction,^64^ were reduced at the time of the EAE peak, as compared to the state prior to immunization. In contrast, potentially beneficial bacteria were enriched at the time of the EAE peak in animals treated with MDSC-PGE2. Thus, coprostanol-forming *Eubacterium coprostanoligenes* group,^65^ as well as *Ruminococcaceae UCG-014* and *Ruminoccus 2*, were previously shown to stimulate lactic acid metabolism toward the production of SCFAs, thus supporting the intestinal barrier.^62^ This is in line with the significantly higher expression of genes controlling tight junctions between enterocytes and the higher level of ALP in fecal samples collected from animals treated with MDSC-PGE2, pointing to the potential of these cells to protect the intestinal epithelial barrier. Also, *Lb. reuteri* and *Ruminoccus 1* were identified by LEfSe analysis as microbial discriminatory markers of the MDSC-PGE2 group at the time of EAE peak. *Lb. reuteri* is a commensal intestinal Firmicute that is highly abundant in the gastrointestinal tract and has been reported to suppress the production of pro-inflammatory cytokines by intestinal epithelial cells^66^ and monocytes,^67^ in addition to reducing intestinal inflammation in different rodent models.^68^ These results indicate that the application of MDSC-PGE2 maintains gut microbiota diversity, prevents disease-induced dysbiosis, and potentially shifts the balance toward microbiota members with immunoregulatory properties.

Considering the important role of SFB in promoting Th17 differentiation and production of IL-17 in the gut, which can lead to EAE development,^69^ it is important to emphasize the result showing that both MDSC types reduced SFB levels in rat stool samples in comparison to the control EAE group.

When considering the predicted microbial metabolic pathways involved in the synthesis of metabolites with well-known effects in the host, S-adenosyl-L-methionine (SAM) cycle I, the precursor of methionine, was shown to be a potential marker pathway in the EAE control group and L-methionine biosynthesis in the MDSC-treated group. The potential enrichment of pathways leading to SAM synthesis in both experimental groups with pronounced EAE symptoms (control and non-efficient MDSC treatment), and not in animals treated with efficient MDSC-PGE2, is in accordance with the studies pointing to the requirement of external methionine and SAM in the stimulation of Th1/Th17 activation and function, as well as in EAE development.^70^ On the other hand, different TCA pathways and glyoxylate bypass pathways,^71^ involved in succinate production and pathways involved in the synthesis of acetate and lactate (glycolysis III and pyruvate fermentation to acetate and lactate II) are shown as potentially dominant microbial marker pathways in MDSC and MDSC-PGE2 groups, respectively. Considering that succinate, the dominant precursor of microbial pyruvate and acetate, belong to SCFAs with previously described anti-inflammatory effects, the enrichment of these pathways could be a beneficial effect of MDSC applications. SCFAs (acetate, propionate, and butyrate) are produced by different bacterial species during dietary fiber fermentation in the large intestine. SCFAs are known to impact gut homeostasis by stimulation of transforming growth factor (TGF)-β production in epithelial cells, induction of Tregs by epigenetic modifications of the Foxp3 locus, stimulation of IL-10 and retinoic acid production by DCs and MFs, thus maintaining immune tolerance.^72^ Also, putrescine and spermidine, the most common polyamines produced by gut microbiota, are synthesized through the super-pathway of polyamine biosynthesis II (POLYAMINSYN3-PWY) and polyamine biosynthesis I (POLYAMINSYN-PWY), which are potentially enriched in the microbiota of MDSC- and MDSC-PGE2-treated animals, respectively. A recent study has reported the immunoregulatory role of spermidine in EAE treatment. After the administration of spermidine in mice with EAE, this polyamine suppressed disease progression by inhibiting MF activity.^73^ Luminal putrescine is important for colonocyte proliferation and maintenance of gut mucosal homeostasis,^74^ which could be in line with our results that both MDSC and MDSC-PGE2 applications increased claudin and occludin expression, thus potentially providing increased protection of the intestinal barrier from systemic inflammation in EAE and MS. These predictions of microbial metabolic pathways were additionally strengthened by HPLC measurement of SCFA and polyamine levels in fecal samples at the peak of EAE. The capacity of MDSC-PGE2 to potentiate the production of propionic acid, butyric acid, and putrescine could be the mechanism of intestinal barrier protection and prevention of further disturbance of the gut microbiota. However, the detailed mechanisms by which different metabolites mutually regulate gut barrier function and the gut immune system, especially in MDSC-based therapies, are still unknown and require further studies. Considering the previously described role of predicted microbial pathways, we could assume that the enrichment of beneficial anti-inflammatory pathways involved in polyamine and SCFA synthesis in the microbiota of MDSC-PGE2 treated animals, could have contributed to the protective effects of this treatment in EAE animals. This study highlights potential new supplements for *in vitro* protocols for immunoregulatory cell-based therapy preparations. Importantly, supplementation of MS patients with these microbial products (SCFAs and polyamines) or diet changes, leading to the enrichment of bacteria producing these metabolites (fiber-rich diet), could additionally improve the effects of MDSC therapy. Although some of these pathways are potentially enriched in the MDSC group, the enrichment of potentially harmful methionine synthesis pathway in this group, as well as in control animals, could contribute to reduced clinical benefits of the therapy in EAE.

In summary, we demonstrated in an EAE model in DA rats that MDSCs activated with PGE2 during *in vitro* differentiation can ameliorate EAE severity, unlike non-activated MDSCs, which could be harnessed to develop MDSC-based therapy for MS in future studies. This research extends our knowledge of host–microbiota interactions in EAE and, for the first time, shows a correlation between gut microbiota composition and the efficacy of MDSC in the amelioration of EAE. Additionally, our results indicated that SCFAs and polyamines are important microbial metabolites in reducing the symptoms of EAE, which could be relevant for the improvement of therapies for MS, either via SCFA- and polyamine-producing probiotics or SCFA- and polyamine-supporting diets.

## 4. Materials and methods

### 4.1. Animals

Dark Agouti (DA) rats were housed in the animal facility at the Institute for Application of Nuclear Energy (INEP), University of Belgrade, according to institutional procedures. All experimental procedures were approved by the Ethical Committee INEP and were in accordance with EU directive 2010/63/eu on the protection of animals used for scientific purposes and approved by the Ministry of Agriculture and Environmental Protection, Republic of Serbia (Decision No. 323–07-11,160/2019-05/1).

### 4.2. Cells

BM cells were isolated from the femurs and tibia of DA rats, after euthanasia with an anesthetic overdose (xylazine/ketamine), as described previously.^75^ After collecting cells in Petri dishes and cell cluster fragmentation in RPMI medium (Sigma-Aldrich Inc., St. Louis, MO, USA), the suspension was filtered in a tube, centrifuged at 1600 rpm for 10 min and washed. The cell precipitate was treated with a lysis buffer (0,8% NH_4_Cl, pH 7,3) for 4 min, followed by centrifugation and washing. Cells were resuspended in 2 mL of complete RPMI medium containing 10% FCS (Gibco/Thermo Fisher Scientific, Waltham, MA, USA) and antibiotic/antimycotic solution (Capricorn Scientific, Ebsdorfergrund, Hessen, Germany) prior to cell counting using Trypan blue dye. For the generation of BM-derived MDSCs, the cells were plated in complete RPMI medium (20 × 10^6^/cells in 12 mL of T75 flasks, Sarstedt, Nümbrecht, Germany) supplemented with recombinant murine (rm) FLT3 (20 ng/mL, Peprotech, Rocky Hill, NJ, USA), rmGM-CSF (20 ng/mL, R&D Systems, Minneapolis, MN, USA), and 40 ng/mL rmIL-6 differentiation cocktail, according to previous protocols.^13,21,22^ For induction of MDSC-PGE2, FLT3/GM-CSF/IL-6 cocktail was additionally supplemented with 1 µg/mL PGE2,^14^ and maintained under 5% CO_2_ and 37°C for 4 days. Additionally, 3 mL of RPMI medium was added on the second day of differentiation. After differentiation, the cells were collected and washed twice with 0.02% NaEDTA in PBS. To enrich low-density MDSC fractions, according to previous protocol,^20^ the cells were placed on Pancoll Density Gradient (1.077 g/mol, PAN Biotech, Aidenbach, Germany) and centrifuged at 4000 rpm for 20 min at room temperature (22°C). Less than 15% of the cells (mostly differentiated granulocytes and dead cells) were found in pellets, whereas low-density fractions were retained as MDSCs. For the generation of BM-derived MFs/DCs, BM cells were differentiated in rmGM-CSF (20 ng/mL) and rmFLT-3 (20 ng/mL) supplemented with a complete RPMI medium for 6 days, according to previous protocols,^28,29^ followed by a density gradient purification step as described for MDSCs.

Splenocytes were isolated from the spleens of euthanized DA rats. The spleen capsule was opened on top, and the tissues were squeezed with a needle, dislodged with a 20 G syringe, filtered through 30 µm filters, and washed in 2% FCS/RPMI medium. The cell pellet was resuspended in 2% FCS/RPMI medium, placed on a Pancoll Density Gradient, and centrifuged at 4000 rpm for 20 min at room temperature. The cells were collected and washed in 2% FCS/RPMI medium. The cell pellet was lysed with lysis buffer for 3 min, and the cells were washed twice before resuspension in complete RPMI medium and counted using Trypan blue.

### 4.3. Proliferation assay

The suppressive properties of BM-derived MDSCs were evaluated in co-culture with ConA-stimulated splenocytes. Splenocytes were first labeled with cell-trace FarRed (Invitrogen, Carlsbad, CA, USA), according to the manufacturer’s instructions, stimulated with ConA (0.5 µg/ml, Sigma-Aldrich Inc.), and then placed in a 96-well plate (3 × 10^5^/well/100 µL). BM-derived MDSCs (6 × 10^4^, 3 × 10^4^, 1.5 × 10^4^, and 0.75 × 10^4^/well/100 µL) were added immediately afterwards, thus providing 1:5, 1:10, 1:20, and 1:40 MDSCs:splenocyte ratios, respectively, and the co-cultures were incubated for 4 days at 37°C, 5% CO_2_, and 90% humidity. Control cultures contained ConA-treated FarRed-labeled splenocytes without MDSCs (biological controls) and technical controls contained ConA-treated non-labeled and non-treated FarRed-labeled splenocytes, cultivated similarly. After culturing, the cells were collected, labeled with PI (5 µg/mL), and analyzed using flow cytometry (BD LSR II, BD Biosciences, San Jose, CA, USA). Cell proliferation was evaluated by analyzing the FarRed dilution after the exclusion of doublets and PI^+^ (dead) cells.

### 4.4. EAE induction and MDSC application

The capacity of MDSCs to suppress inflammation *in vivo* was evaluated on a model of EAE described previously.^18^ Briefly, female DA rats eight-week-old, with body weights ranging from 138 to 166 g, were randomly divided into three groups, each group containing 10 animals weighted similarly. DA rats were anesthetized with ketamine/xylazine and then immunized with spinal cord homogenate (50% w/v in saline) emulsified in complete Freund’s adjuvant (CFA) containing 4 mg/ml *Mycobacterium tuberculosis* (Difco/BD Diagnostics, Sparks, MD, USA) administered to the right-hind footpad. One day after immunization, one group received BM-derived MDSCs (2 × 10^6^ cells/rat) and one group received the same number of MDSC-PGE2 intraperitoneally. From day 8 post-immunization, the animals were weighed and assessed for signs of the disease daily and scored according to the following scale:0, no clinical signs; 1, flaccid tail; 2, hind limb paresis; 3, complete bilateral hind limb paralysis often associated with incontinence; and 4, moribund state or death. Intermediate scores were assigned if neurological signs were of lower severity than typically observed. Several disease parameters were examined to evaluate the severity of EAE, including incidence, mean day of onset, duration of illness, mean maximal severity score, and cumulative disease index (the sum of the daily mean clinical scores for a group over a given number of days). Animals were observed for 27 days after immunization. All experiments were repeated twice.

Fecal samples were collected before EAE induction (0^th^ day) and at the peak of the disease (15^th^ day) and stored immediately at −80°C. At the peak of the disease, the animals in each group were deeply anesthetized and perfused with physiological solution via hearth’s right chamber for 5 min, and then sacrificed for organ collection. Three additional healthy, non-immunized, and non-treated animals of similar age/weight as the experimental groups were sacrificed in the same manner and used for intestinal sample collection. The small intestines were isolated and stored at −80°C, and the spinal cord and spleens were processed for FACS analysis and qPCR. Splenocytes and spinal cord infiltrating cells were isolated as described previously,^76^ with collagenase (0.5 mg/mL, Gibco) and DNAse (40 IU/mL, Sigma) digestion and Percoll density gradient separation (4000 rpm, 20 min).

### 4.5. MDSC tracking analysis

MDSC-PGE2 differentiated *in vitro* and purified by density gradient were labeled with CFSE (0.5 µM, Thermo Fisher Scientific) according to the manufacturer’s protocol, and then administered intraperitoneally to rats that were either immunized the day before (n = 9) or non-immunized (n = 9). For tracking analysis of CFSE-labeled MDSC-PGE2, the animals were euthanized 3, 7, or 10 days after MDSC-PGE2 administration, and the organs were collected for analysis by flow cytometry. Payer’s patches and inguinal, axillary, and mesenteric lymph nodes were minced through steel mashes and filtered through 30 µm pore filters, followed by washing twice in 2% FCS/RPMI. Splenocytes and spinal cord-infiltrating cells were isolated as described in Section 4.4. After isolation, the cells were washed in PBS, labeled with fixable viability dye (FVS620, BD Biosciences) according to the manufacturer’s protocol, and then labeled with CD45:PE and CD11b: biotin-streptavidin PE/Cyanin7 for flow cytometry analysis.

### 4.6. Flow cytometry

Immediately after MDSC *in vitro* differentiation and tissue collection at the peak of disease (15^th^ day post immunization), single-cell suspensions were prepared, stained, and characterized on a BD LSR II flow cytometer (BD Biosciences) using FlowJoVX (BD Biosciences) or FCS Express 7 (Denovo software). The cells were washed once in PBS containing 0.01% sodium azide (Sigma-Aldrich), incubated with 2% normal rat serum (eBioscience, Frankfurt am Main, Germany) for 15 min, and then labeled with fluorochrome-conjugated monoclonal antibodies. For surface staining, cells were incubated with following antibodies diluted in recommended concentrations in PBS/0.01% NaN_3_: mouse IgG1 negative control-Alexa Fluor 488 (F8-11-13), mouse IgG1 negative control-phycoerythrin (PE) (F8-11-13), anti CD68-Alexa Fluor 647 (ED1), anti-CD4-biotin (clone W3/25), anti-MHC class II-RPE (OX-6), anti-CD172a-biotin (OX-41, SIRP-α), anti-CD11b-fluorescein isothiocyanate (FITC) (OX-42), anti-CD3-Alexa Fluor 647 (1F4) (all from Bio-Rad, Hercules, California, USA); mouse IgG1 negative control (MOPC-21)-Alexa Fluor 647, mouse IgG1 negative control-biotin (AB_2550616), anti-IL-17A-FITC (eBio17B7), anti-Granulocytes antibody-FITC (HIS48), anti-CD161-Biotin (10/78) (all form Thermo Fisher Scientific, Waltham, Massachusetts, USA); anti-CD25-FITC (OX-39), anti-CD103-biotin (OX-62), anti-IL-10-PE (A5-4), anti-RP-1-Alexa Fluor 647 (RP-1), anti-CD86-biotin (24 F) (all form BD Pharmingen, San Diego, California, USA); mouse IgG1 kappa negative control-Allophycocyanin (APC) (P3.6.2.8.1), anti-FoxP3-APC (FJK-16s) (all from eBioscience); anti-IFN-γ-FITC (DB-1), anti-CD11b-APC (OX42), anti-CD86-FITC (24 F), anti-CD45-PE (OX1), anti-CD11b-biotin (OX42), anti-CD80-PE (3H5), PerCP/Cyanine5.5 Streptavidin, PE/Cyanine7 Streptavidin, (BioLegend, San Diego, California, USA); rabbit polyclonal anti-mouse IgG (whole molecule)-FITC (Sigma-Aldrich). The incubation period lasted 30 min at 4°C.

Intracellular staining of cells was conducted after surface staining using a flow cytometry fixation and permeabilization kit I (R&D Systems). Intracellular staining of IFN-γ, IL-10, and IL-17 in splenocytes and spinal cord infiltrating T cells was carried out after 6 h activation of cells with phorbol-12-myristate-13-acetate (PMA, 20 ng/mL) and ionomycin (500 ng/mL) in the presence of monensin (2 µM) (all from Sigma-Aldrich). For each analysis, more than 5,000 cells were gated according to their specific side scatter (SSC)/forward scatter (FSC) properties, thereby avoiding cells with low SSC/FSC properties (predominantly dead cells), as indicated. Alternatively, FVS620 viability dye was used to exclude dead cells. Signal overlap between the channels was compensated before each experiment using single-labeled cells, and non-specific fluorescence was determined using the appropriate negative controls.

### 4.7. Light and epifluorescence microscopy analyses

MDSCs and MFs/DCs differentiated from BM cells, as described in Section 4.2, were prepared as cytospins (1 × 10^4^ cells/70 µL PBS) using a Rotafix 32 Centrifuge (Andreas Hettich GmbH & Co., Tuttlingen, Germany), air-dried prior to staining with May-Grunwald Giemsa (Sigma Aldrich), and analyzed by light microscopy.

To evaluate cellular infiltrates in the spinal cords of animals with EAE, the spinal cords were isolated carefully from euthanized animals at the peak of the disease (15^th^-day post-immunization), and the lumbar part of the spinal cord was fixed in 4% paraformaldehyde for two weeks. Tissues were dehydrated in a series of ethanol (70%, 96%, 96%, and 100%) and xylol (33% in ethanol, 66%, 100%, and 100%) solutions, and then paraffin-embedded using the Tissue Embedding Center EC 350 (Especialidades Médicas Myr, Spain). Cross-sections 5–7 µm thick) were made using a manual microtome, and the slides were rehydrated in dilutions of xylol in PBS (100%, 96%, and 70%) prior to staining. Hematoxylin and eosin staining was performed using the corresponding dyes purchased from Sigma Aldrich, embedded in Canada balsam, and analyzed by light microscopy.

For immunofluorescence analysis, rehydrated paraffin cross-sections were blocked with BSA for 1 h, and then incubated with mouse-anti rat CD45 (OX22, BioLegend, 5 µg/section) and polyclonal rabbit-anti rat MBP (Novus Biologicals, 5 µg/section) at 4°C overnight, washed in PBS, and then incubated for 3 h with the secondary antibodies anti-mouse IgG-Alexa 546 and anti-rabbit IgG-Alexa 488 (both 2 µg/section, Thermo Fisher). After washing in PBS, the sections were stained with DAPI (1 µg/section), embedded in mounting medium (Bio-Optica, Milano, Italy), and analysed using an epi-fluorescent microscope (Zeiss Axiovert A1) with a UV filter set for DAPI (UV-2B, ex:330–380 nm, DM 400, BA 435), green filter set for detection of MBP (B-2A, ex:450–490 nm, DM 505, BA 520), and red filter set for detection of CD45 (G-2A, ex:510–560 nm, DM 575, BA 590). The images taken by each filter set were acquired as monochromatic and later merged offline using ImageJ software (National Institutes of Health, Bethesda, Maryland, USA).

### 4.8. Measurement of Cytokines and NO

For cytokine measurements, at the disease peak, isolated splenocytes were placed in 24-well plates (2 × 10^6^ cells/well/1000 µL) (Sarstedt) and stimulated with ConA (2.5 µg/mL, Sigma-Aldrich) for 24 h. Cell- and debris-free culture supernatants were collected and assayed for IL-1β, TNF-α, IL-6, IL-4, IL-17, IFN-γ, and IL-10 using enzyme-linked immunosorbent assay (ELISA) according to the manufacturer’s instructions (all from R&D Systems). NO was measured in MDSC culture supernatants on day 4 of cultivation using Griess reagent (Abcam, Cambridge, UK), according to the manufacturer’s instructions. Absorbance was measured at λ = 540 nm (BioTek EL800, Agilent Scientific Instruments, CA, USA), and the concentration was determined from the standard curve.

### 4.9. Quantitative Real-Time PCR (qPCR)

Phenol/chloroform extraction of RNA from intestinal samples was performed as previously described.^77^ Briefly, total RNA extraction was performed using liquid nitrogen homogenization of tissue, followed by resuspension in 500 μl denaturing solution on ice. The next step included acid phenol (pH = 4) extraction, which was repeated twice, and RNA precipitation with isopropanol.

For RNA isolation from mononuclear cell fractions, cells were resuspended in 500 μL of TRIzol (Thermo Fisher Scientific) with the addition of 100 μL of chlorophorm (Sigma-Aldrich, Merck) and centrifuged at 12,000 g for 5 min at 4°C. The upper aqueous phase was transferred to a new RNase-free tube and mixed with 600 μL of cold 70% ethanol. Further steps were performed using the Total RNA Purification Mini Spin Kit (Genaxxon Bioscience, Bieberach, Germany) according to the manufacturer’s instructions with 30 min DNase incubation step. RNA concentrations from all samples were measured on BioSpec-nano (Shimadzu, Tokyo, Japan) and RevertAid RT Reverse Transcription Kit (Thermo Fisher Scientific) was used for cDNA generation from 0.5 μg and 0.1 μg of isolated total RNA, for intestine and mononuclear cell samples, respectively. Target gene expression was determined using the 7500 real-time PCR system (Applied Biosystems, Foster City, CA, USA) using the FastGene 2X IC Green Universal ROX (Nippon Genetics, Tokyo, Japan) protocol. Normalization was performed against the rat *Actb* gene and bacterial 16S rRNA gene, and the results were expressed as relative target abundance using the 2^−ΔΔCt^ method. The levels of claudin and occludin mRNA in the experimental groups were calculated as fold-changes in the levels of claudin and occludin in healthy animals (relative mRNA expression = 1).

Primers used in this study are listed in Table 1. All the primers were obtained from Thermo Fisher Scientific.

**Table 1.** List of gene primers used in this study.

Primers	Primer Sequences (5′-3′)
*Actb* Forward	AGCCATGTACGTAGCCATCC
*Actb* Reverse	CTCTCAGCTGTGGTGGTGAA
*Cldn5* Forward	GAACTACGTCTAAGGGCGGG
*Cldn5* Reverse	ACCCAACCTAACTTGCCTCG
*Ocln* Forward	TCATGCCTTGGGGATTGAGC
*Ocln* Reverse	AGGACTTCCCAGAGTGCAGA
*Arg1* Forward	CAAGCTGGGAATTGGCAAAG
*Arg1* Reverse	GGTCCAGTCCATCAACATCAAA
*Ptgs2* Forward	TCCAGTATCAGAACCGCATTGCCT
*Ptgs2* Reverse	AGCAAGTCCGTGTTCAAGGAGGAT
*Nos2* Forward	GACCAGAAACTGTCTCACCTG
*Nos2* Reverse	CGAACATCGAACGTCTCACA
*Bact 1369* Forward	CGGTGAATACGTTCCCGG
*Prok 1492* Reverse	TACGGCTACCTTGTTACGACTT
SFB-specific 16S rRNA gene Forward	AGGAGGAGTCTGCGGCACATTAGC
universal 16S rRNA gene Reverse	TCCCCACTGCTGCCTCCCGTAG

### 4.10. Fecal Alkaline Phosphatase (ALP) activity

ALP activity, as a measure of intestinal barrier integrity, was determined in fecal samples collected from the three groups of animals at the peak of EAE (15 dpi) according to Ismael et al.^78^ Each sample (35 mg per sample) was homogenized with 1750 µL of extraction buffer (1 mM MgCl_2_ and 10 mM Tris-HCl, pH 8.0) and centrifuged at 10,000 g for 20 min. The supernatants were mixed at a ratio of 1:1 with dilution buffer (1 mM MgCl_2_ and 200 mM Tris-base, pH 10.4) and incubated for 10 min at 37°C in a heating block. Nitrophenylphosphate at a final concentration of 5 mM was added to the mixture and incubated for an additional 5 min in the heating block. The reaction was stopped on ice with cold 0.02 M NaOH. The absorbance was measured at 410 nm. The protein content of the fecal samples was determined using the Pierce BCA Protein Assay Kit (Thermo Fisher, Lisboa, Portugal) according to the manufacturer’s specifications, and the fecal ALP activity was expressed as mU ALP/mg of protein.

### 4.11. HPLC-UV quantitative determination of selected fecal metabolites

All HPLC experiments were performed using an UltiMate 3000 UHPLC system (HPLC-UV) (Thermo Scientific, Breda, the Netherlands). Data processing was performed using the Chromeleon version 6.8 software (Thermo Fisher Scientific, MA, USA). Fecal SCFA extraction and HPLC quantification were performed as previously described.^79^ a Hypersil Gold column (150 × 4.6 mm i.d.) with a particle size of 3 μm (Thermo Scientific, Waltham, MA, USA) was used for chromatographic separations and protected by a guard column. The mobile phase consisted of 20 mM NaH_2_PO_4_ (Serva, Heidelberg, Germany) with pH adjusted to 2.22 using H_3_PO_4_ (Merck, Darmstadt, Germany), and HPLC-grade acetonitrile (Sigma-Aldrich, St. Louis, Missouri, USA). Gradient elution was performed, as shown in Table 2. The temperature in the column compartment was maintained at 30°C during chromatographic separation. The injection volume for each sample was 10 μL. The UV detector was set at a wavelength of 210 nm.

**Table 2.** Elution gradient in chromatographic separation of fecal SCFAs.

Retention (min)	Flow (mL/min)	NaH_2_PO_4_ (%)	ACN (%)
3.5	0.8	100.0	0.0
4	0.4	92.5	7.5
7.5	0.4	92.5	7.5
8	0.4	85.0	15.0
10.5	0.4	85.0	15.0
11	0.4	80.0	20.0
13	0.4	80.0	20.0
13.5	0.4	75.0	25.0
20	0.4	75.0	25.0
20.5	1	100.0	0.0

The external standard calibration curve method was used to quantify acetic, propionic, and butyric acids. Succinic acid was used as an internal standard to compensate for the variability in sample preparation. SCFA standard (Sigma-Aldrich, St. Louis, Missouri, USA) solutions were prepared at concentrations ranging from 0,5 mM to 25 mM for each of the SCFAs measured.

Rat fecal samples were diluted 11-fold by the addition of 10-fold weight of 10 mM phosphate-buffered saline (PBS) pH 7.38 containing 150 μM 1,6-diaminohexane (Sigma-Aldrich, St. Louis, Missouri, USA) as an internal standard. Polyamines were extracted by vortexing for 1 min, followed by incubation on ice for 20 min. The suspension was centrifuged (15,000 g for 10 min) to obtain the supernatant. Fecal extracts were stored at −20°C until analysis.

The derivatization of polyamines and HPLC quantification were performed according to the method described by Redmond et al.^80^ with minor modifications. A Luna C18(2) column (250 × 4.6 mm i.d.) with a particle size of 5 μm and pore size of 100 Å (Phenomenex, Torrance, California, USA) was used for chromatographic separations. The mobile phase used was methanol/water (58:42). The chromatography was performed under isocratic conditions. The temperature in the column compartment was maintained at 27°C during chromatographic separation. The injection volume for each sample was 20 μL. The UV detector was set at a wavelength of 227 nm.

The external standard calibration curve method was used for quantification of putrescine and spermidine. Putrescine and spermidine standard solutions were prepared at concentrations ranging from 2 μM to 400 μM.

### 4.12. DNA extraction and sequencing

Genomic DNA extraction was performed on all fecal samples at once to avoid batch effects using the ZR Fecal DNA MiniPrep Kit (Zymo Research, Orange, CA, USA) following the manufacturer’s instructions. The concentration of total DNA isolated from rat fecal samples was measured using a BioSpec-nano (Shimadzu) and Qubit fluorometer (ThermoFisher/Invitrogen). All samples were stored at −20°C until sequencing and qPCR for SFB detection. 16S rRNA-based metagenomics targeting the V3-V4 region was conducted at Novogene (Beijing, China) on the Illumina NovaSeq 6000 platform in PE250 mode using 341 F/806 R as a primer set. The Phusion High-Fidelity PCR Master Mix (New England Biolabs, Ipswich, MA, USA) was used for all PCR reactions, and the resulting amplicons were detected on a 2% agarose gel using SYBR green. For further experiments, samples with band between 400 and 450 base pairs were chosen. Equimolar ratios of PCR products were mixed and purified using a Qiagen Gel Extraction Kit (Qiagen, Hilden, Germany). The NEBNext Ultra DNA Library Prep Kit for Illumina was used for library preparation, followed by quantification using a Qubit fluorometer (Thermo Fisher Scientific) and sequencing.

### 4.13. Microbiota data processing and analysis

Paired-end (PE) reads were assigned to the samples based on their unique barcodes and then truncated by cutting off the barcode and primer sequences. Demultiplexed raw reads with primers and barcode sequences removed were imported into QIIME2 v2020-8,^81^ and their quality was checked. After observing interactive quality plots output from demux summarize plugin and choosing trimming parameters, DADA2 pipeline^82^ was performed with following parameters “–p-trunc-len-f 227” and “–p-trunc-len-r 224”. Denoising and chimeric sequence filtering resulted in the merging of high-quality paired-end reads and the generation of a feature table with 2971 features and corresponding representative sequences. Taxonomic assignment was performed using the naïve Bayes classifier plugin on reference sequences extracted from the SILVA 132 database (99% identity criterion) and classify-sklearn plugin.

QIIME2 artefacts and metadata files were imported into RStudio (v1.4.1717) (RStudio Team) with the qiime2R package functions^83^ and merged into phyloseq objects^84^ for downstream analysis. To obtain reproducible results, we used the set.seed function with seed = 999, followed by phyloseq (v1.36.0) functions for the alpha (Shannon and Simpson indices) and beta diversity (unweighted UniFrac distances) metrics calculation with the rarefying sample size set at 21,600. Statistical significances for alpha and beta diversity indices between the 0 dpi and EAE groups, as well as between unmatched observations, were examined using paired Welch Two Sample *t*-test and unpaired *t*-test, respectively. Principal coordinates analysis (PCoA) plots based on the Unweighted UniFrac distance matrix were generated to better visualize between-sample differences in microbial communities. Significant differences between group similarities were examined by analysis of similarities (ANOSIM)^85^ using the vegan^86^ package. An ANOSIM R value close to 1 indicates disimilarity between groups.

To identify differentially abundant taxa at the species, genus, and family levels, we employed QIIME2 analysis of composition of microbiomes (ANCOM) pipeline,^87^ and taxa with a W value greater than 1 were additionally tested with Pairwise Wilcoxon test or Paired T test in R. LEfSe^88^ pipeline via the Galaxy framework was used to discover species biomarkers between groups at the peak of disease with a logarithmic LDA score above 3.5, along with an alpha value of <0.05, for the factorial Kruskal-Wallis and all-against-all strategies for multi-class analysis.

Predictive metagenome functions at the peak of disease were obtained using the PICRUSt2^89^ pipeline based on marker gene surveys and the MetaCyc pathways database with default options. To unveil discriminative predicted pathways between these groups, PICRUSt2 output was analysed using LEfSe via the Galaxy framework, and pathways meeting an LDA significance threshold of >2 were visualized.

Normality of the distribution of continuous variables was tested using the Shapiro–Wilk normality test. A *p*-value of 0.05 or lower is taken to indicate statistical significance (* *p* < 0.05, ** *p* < 0.01, *** *p* < 0.001). For multiple comparisons, the Benjamini–Hochberg procedure was applied to adjust the p-values. All plots were computed using graphics (v4.1.0) (R Core Team) and ggplot2 (v3.3.5) packages unless otherwise specified.

The data for this study have been deposited in the European Nucleotide Archive (ENA) (https://www.ebi.ac.uk/ena) under accession number PRJEB52193 and secondary accession number ERP136892.

### 4.14. Statistical analysis

The Student’s *t*-test was used to compare immune marker expression, relative mRNA expression levels, and NO production between MDSC and MDSC-PGE2. One-way ANOVA followed by Tukey’s multiple comparisons test was used to compare the relative mRNA expression levels and immune marker expression/production as well as fecal ALP activity between all groups at the peak of the disease. Statistical significance in clinical scores between time points across the three groups was determined using two-way ANOVA with Dunnett’s *post-hoc* test. Disease severity parameters between the control group and treatments were examined using Student’s *t*-test and are represented as mean±standard deviation (SD). The log-rank test was used to compare the individual clinical scores of EAE between groups. The levels of SCFA and polyamines were compared between the groups using the Kruskal–Wallis test. All the statistical analyses were performed using GraphPad Prism (v9.1.0) (GraphPad Software).

## Supplementary Material

Supplemental Material

## Data Availability

16S rRNA gene sequencing reads generated in this study were deposited in the European Nucleotide Archive (ENA) (Accession codes: PRJEB52193 and ERP136892).https://www.ebi.ac.uk/ena/browser/view/PRJEB52193
