# Supplementary material for: Myeloid-derived suppressor cells prevent disruption of the gut barrier, preserve microbiota composition, and potentiate immunoregulatory pathways in a rat model of experimental autoimmune encephalomyelitis
Source: Gut Microbes. 2022 Oct 2;14(1):2127455. doi: 10.1080/19490976.2022.2127455 (PMC9543149; doi:10.1080/19490976.2022.2127455)
Supplement: Supplemental Material [file KGMI_A_2127455_SM7262.docx]

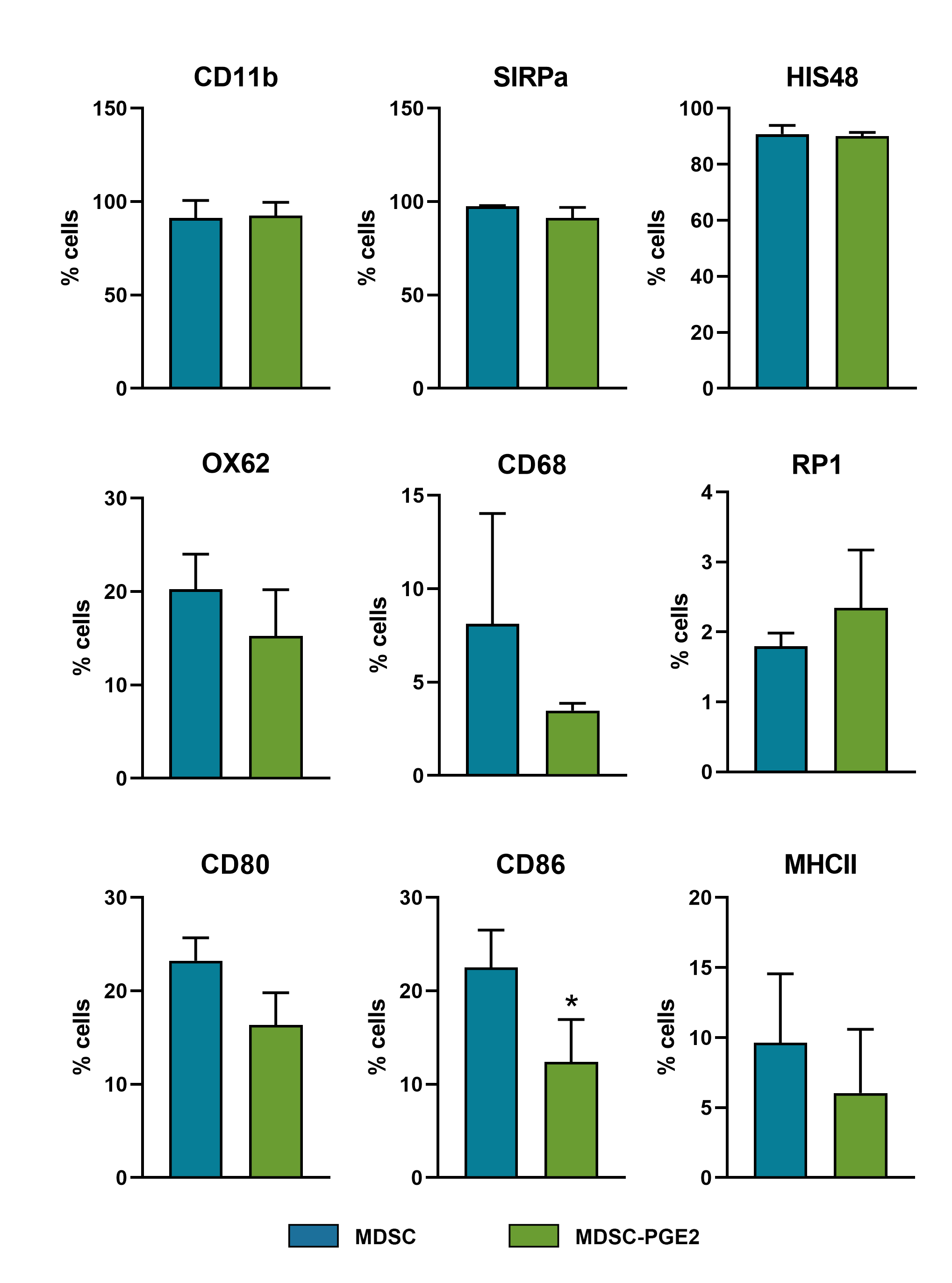


**Supplement Figure 1.** Phenotypic characterization of MDSC. MDSC were differentiated from bone marrow of DA rats in the presence of FLT3/GM-CSF/IL-6 (MDSC, blue) or FLT3/GM-CSF/IL-6 and PGE2 (MDSC-PGE2, green) for 4 days, and the phenotype was analysed by Flow cytometry (representative data are shown in Figure 1). Indicated markers expressed by rat myeloid cells are shown as mean % of positive cells ±SD from three independent experiments. (Student’s t-test, * p<0.05).


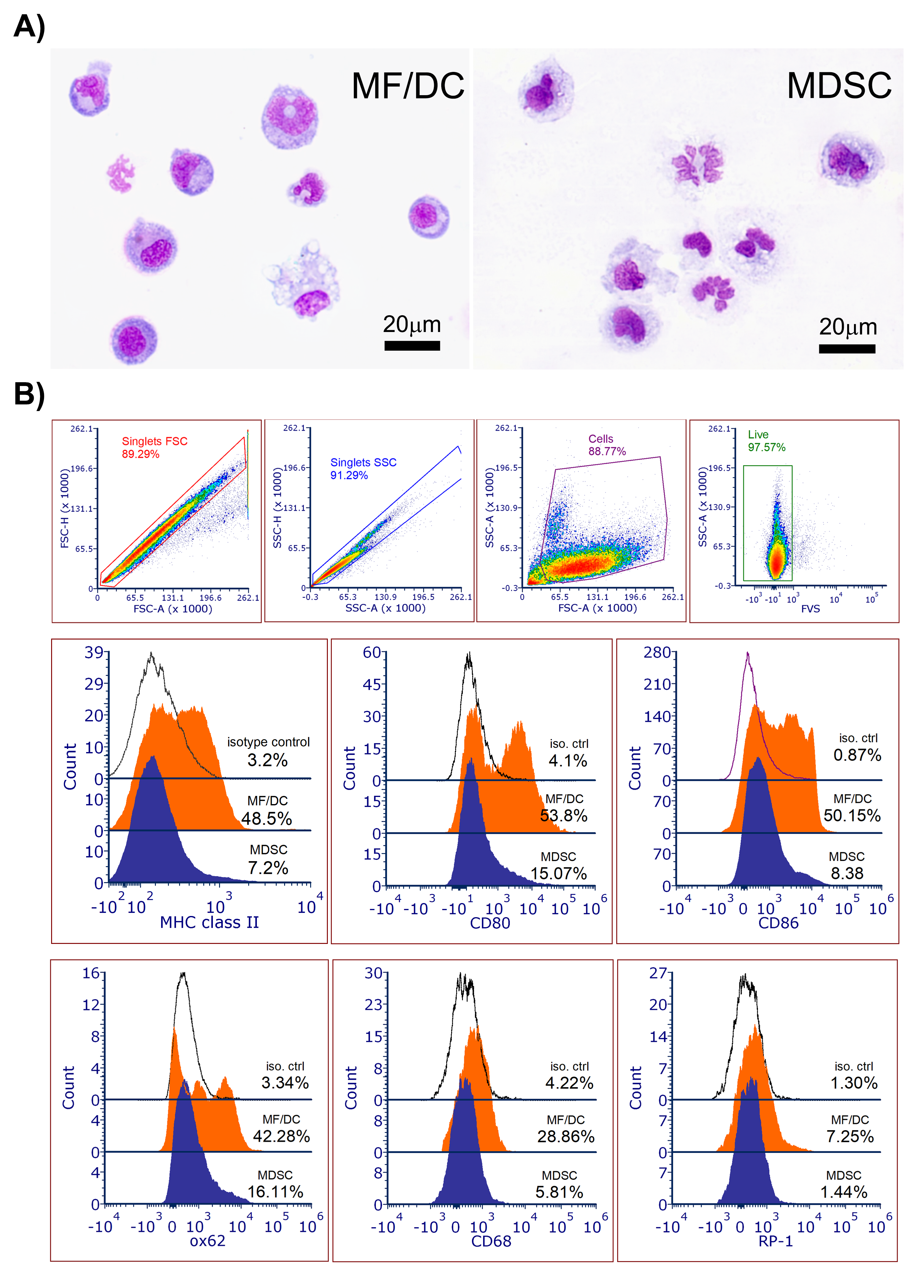


**Supplement Figure 2.** Comparison between MDSC, MF/DC phenotype. **A.** Light microscopy images are shown of the cytospins of MF/DC, differentiated in the presence of FLT3/GM-CSF, and MDSC, differentiated in the presence of FLT3/GM-CSF/IL-6, for 4 days, following staining by May-Grünwald Giemsa. **B.** Representative gating strategy and the phenotype analysis of indicated markers are shown as overlaid histograms of MDSC (blue), MF/DC (orange) and isotype controls (white).

**Supplement Figure 3.** The evaluation of cellular infiltrates in spinal cord of control, MDSC and MDSC-PGE2 EAE induced animals. To evaluate cellular infiltrates in spinal cords in animals with EAE, the lumbar parts of spinal cords were isolated at the peak of the EAE (15^th^-day post-immunization), and paraffin-embedded. 5-7 µm thick cross-sections of spinal cords were stained with Hematoxylin/eosin and by immunofluorescently labeled CD45 and MBP antibodies. Scale bars indicate 50µm.


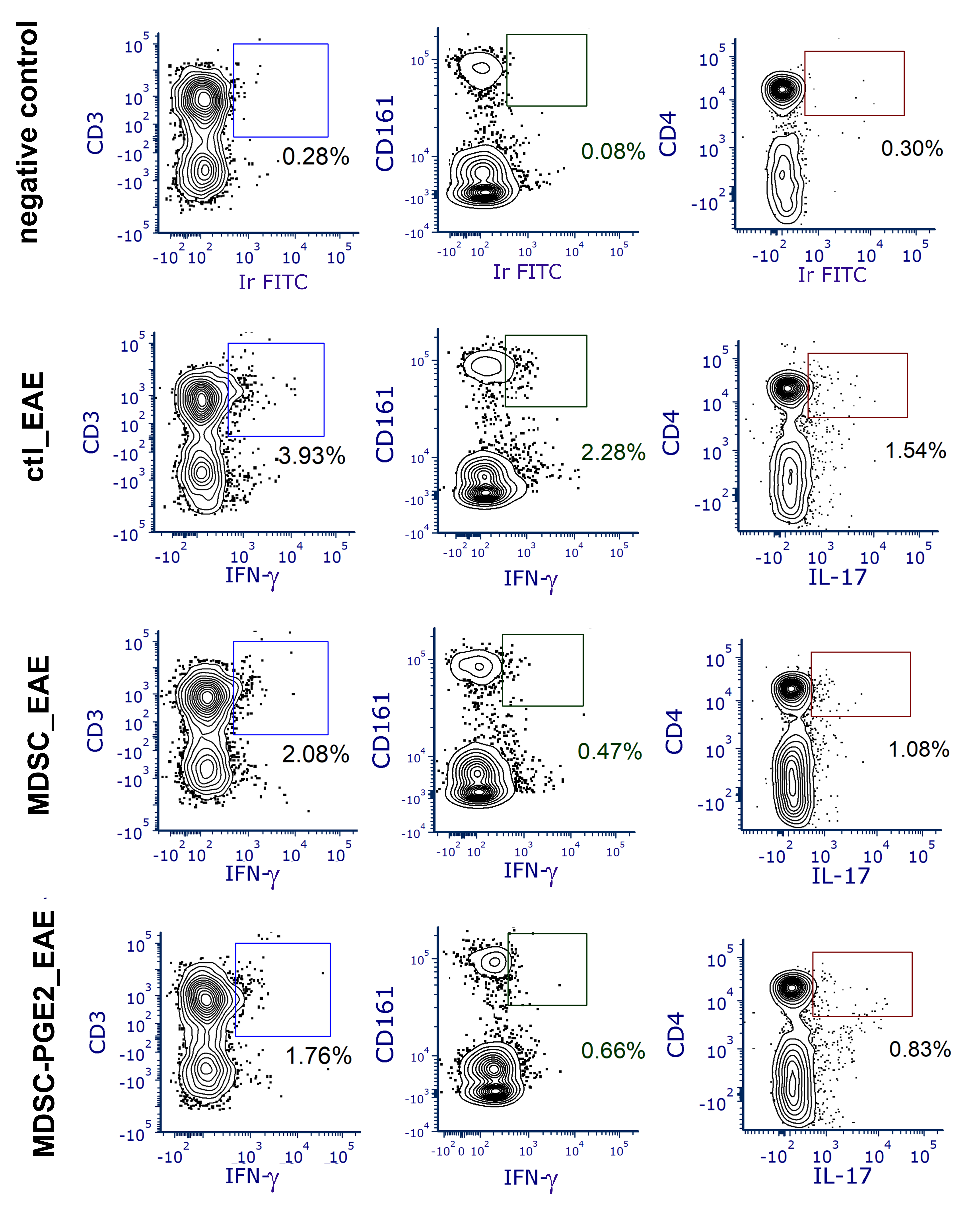


**Supplement Figure 4.** The representative gating strategy for the analysis of interferon (IFN)-γ-producing NK (CD161^+^IFN-γ^+^), Th17 (CD4^+^IL-17^+^) and (IFN)-γ-producing T (CD3^+^IFN-γ^+^) infiltration in target tissues isolated at the peak of EAE from control, MDSC-treated and MDSC-PGE2-treated animals.


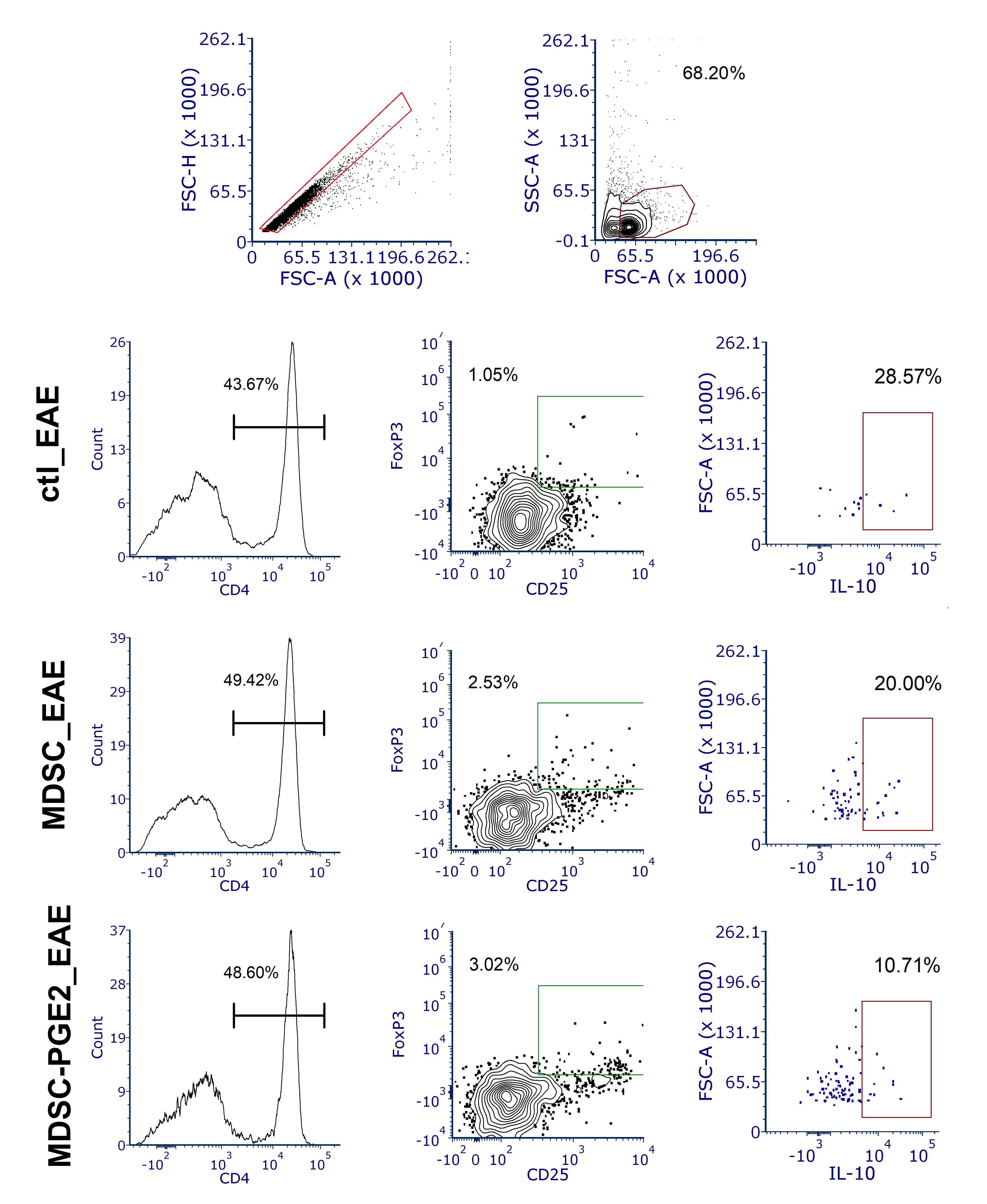


**Supplement Figure 5.** The representative gating strategy for the analysis of regulatory T cells (Tregs) (CD4^+^CD25^+^FoxP3^+^) and IL-10-producing Tregs (CD4^+^CD25^+^FoxP3^+^IL-10^+^) lymphocytes infiltration in target tissues isolated at the peak of EAE from control, MDSC-treated and MDSC-PGE2-treated animals.


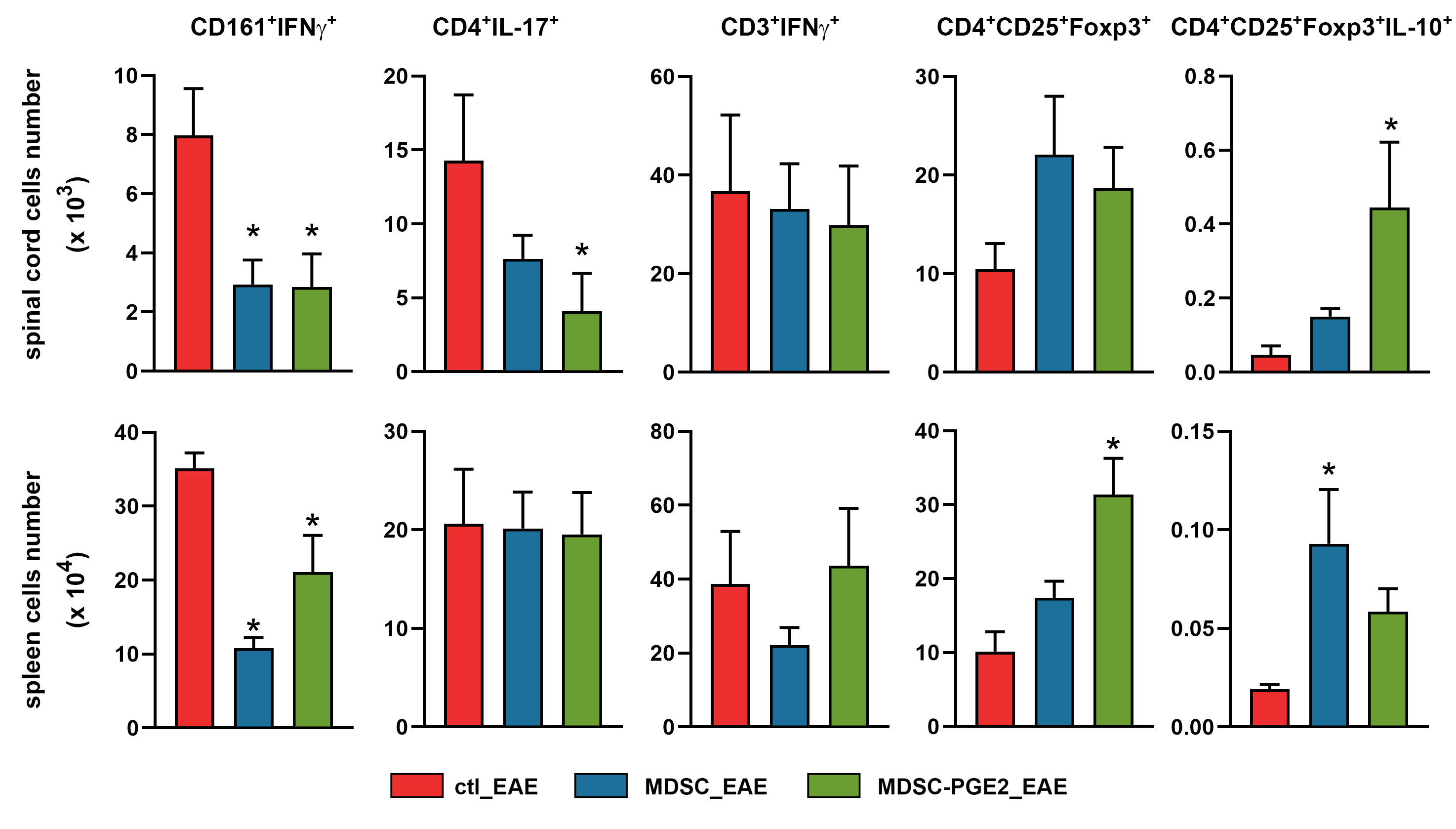


**Supplement Figure 6.** The number of immune cell populations in control animals and animals treated with MDSC and MDSC-PGE2 at the peak of EAE. The number of interferon (IFN)-γ-producing NK (CD161^+^IFN-γ^+^), Th17 (CD4^+^IL-17^+^) and (IFN)-γ-producing T (CD3^+^IFN-γ^+^), regulatory T cells (Tregs) (CD4^+^CD25^+^FoxP3^+^) and IL-10-producing Tregs (CD4^+^CD25^+^FoxP3^+^IL-10^+^) lymphocytes in the spleen and spinal cord samples isolated from animals (n=5 animals in each group) at the peak of EAE (15dpi) in control (ctl_EAE, red) group, and groups treated with either MDSC (MDSC_EAE, blue) or MDSC-PGE2 (MDSC-PGE2_EAE, green), calculate from the percentages of these cell populations obtained by flow cytometry analysis and the live cell count by Trypan blue exclusion assay, after the isolation of cells from the tissues.


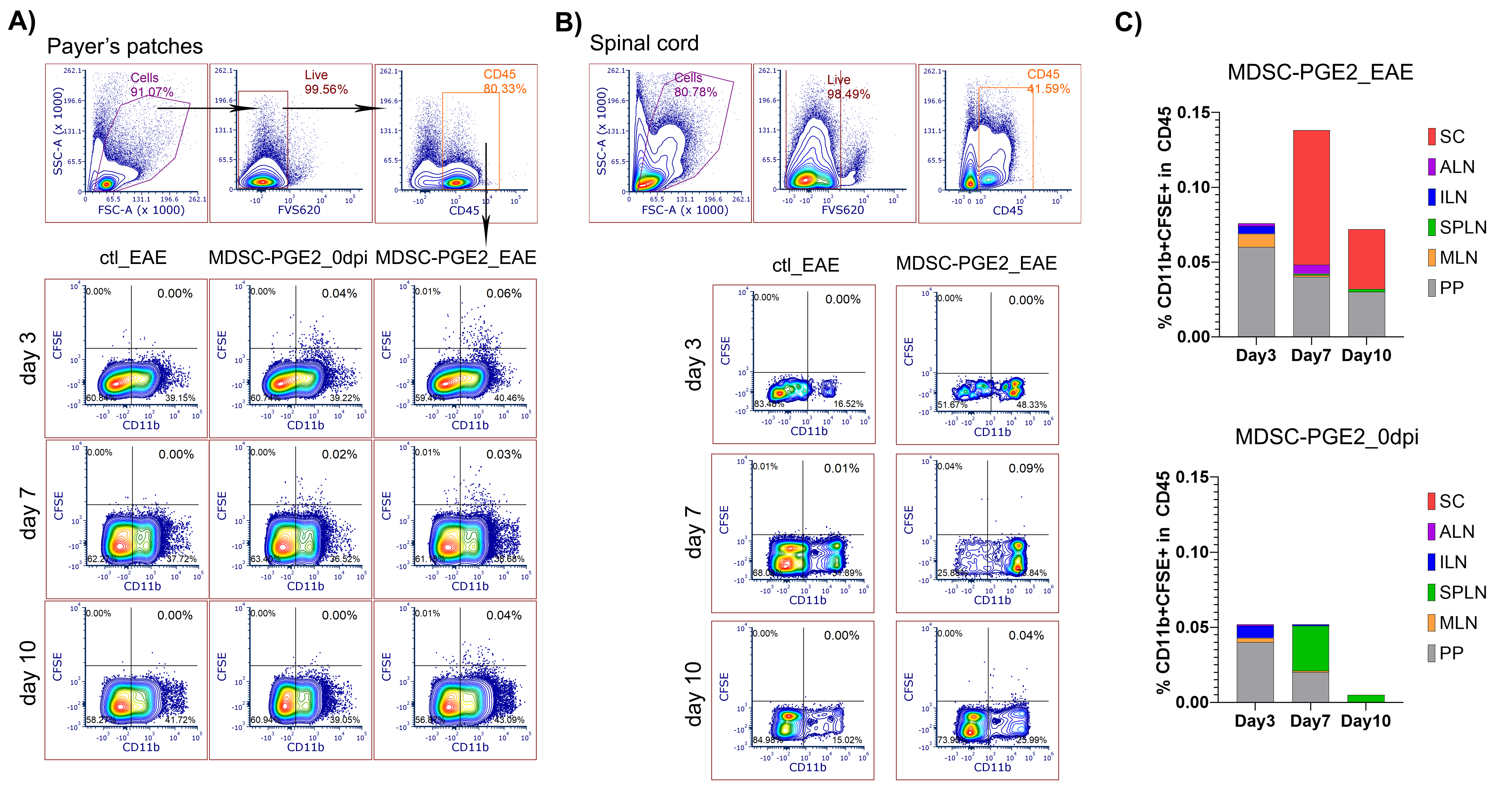


**Supplement Figure 7.** MDSC tracking analysis. MDSC-PGE2 were labelled with CFSE and then administered intraperitoneally into rats that were either immunized the day before to develop EAE (n=9), or non-immunized (n=9). CFSE-labelled MDSC-PGE2 were detected in CD45^+^CD11b^+^ population in Payer’s patches, spinal cord, inguinal, axillary and mesenteric lymph nodes 3, 7 or 10 days after the MDSC administration by flow cytometry analysis. **A.** The representative flow cytometry data are shown for Payer’s patches and **B.** spinal cord, and **C.** bars presenting the percentage of these cells are shown for Payer’s patches, spinal cord, inguinal, axillary and mesenteric lymph nodes.
